# Current and potential contributions of large-scale food fortification to meeting micronutrient requirements in Senegal: a modelling study using household food consumption data

**DOI:** 10.1136/bmjph-2024-001221

**Published:** 2024-11-09

**Authors:** Katherine P Adams, Reina Engle-Stone, Brent Wibberley, Becky L Tsang, Ann Tarini, Maguette Beye, Laura A Rowe

**Affiliations:** 1 Institute for Global Nutrition, University of California, Davis, Davis, California, USA; 2 Department of Nutrition, University of California, Davis, Davis, California, USA; 3 TechnoServe, Arlington, Virginia, USA; 4 USAID Advancing Food Fortification Opportunities to Reinforce Diets, Arlington, Virginia, USA; 5 Food Fortification Initiative, Atlanta, Georgia, USA; 6 Independent Consultant, Laval, Quebec, Canada; 7 Helen Keller International, Dakar, Senegal; 8 Nutrition International, Ottawa, Ontario, Canada

**Keywords:** Public Health, Nutrition Assessment, Nutritive Value

## Abstract

**Introduction:**

Micronutrient deficiencies are common among women of reproductive age (WRA) and children in Senegal. Large-scale food fortification (LSFF) can help fill gaps in dietary intake.

**Methods:**

We used household food consumption data to model the contributions of existing LSFF programmes (vitamin A-fortified refined oil and iron and folic acid-fortified wheat flour) and the potential contributions of expanding these programmes to meeting the micronutrient requirements of WRA (15–49 7 years) and children (6–59 months).

**Results:**

Without fortification, apparent inadequacy of household diets for meeting micronutrient requirements exceeded 70% for vitamin A, thiamin, riboflavin, folate and zinc, was 61% for iron among WRA (43% among children) and was ~25% for vitamin B_12_. At estimated current compliance, fortified refined oil was predicted to reduce vitamin A inadequacy to ~35% and could further reduce inadequacy to ~25% if compliance with the standard improved. Fortified wheat flour at estimated current compliance reduced iron and, especially, folate inadequacy, but improvements in compliance would be necessary to achieve the full potential. Beyond existing programmes, expanding wheat flour fortification to include additional micronutrients was predicted to have a modest impact on thiamin and riboflavin inadequacies and larger impacts on vitamin B_12_ and, especially, zinc inadequacies. Adding a programme to import fortified rice could further reduce inadequacies of multiple micronutrients (generally by >10 percentage points), although potential risk of high intake of vitamin A, folic acid and zinc among children should be carefully considered. With both wheat flour and rice fortification, predicted prevalence of vitamin A, iron and zinc inadequacy remained above 25% in some regions, pointing to the potential need for coordinated, targeted micronutrient interventions to fully close gaps.

**Conclusions:**

When considered alongside evidence on the cost and affordability of these programmes, this evidence can help inform the development of a comprehensive micronutrient intervention strategy in Senegal.

**Key messages**

**WHAT IS ALREADY KNOWN ON THIS TOPIC**

- There is a high burden of micronutrient deficiencies among women of reproductive age and children in Senegal, and evidence is needed on the contributions of large-scale food fortification (LSFF) programmes for meeting micronutrient requirements.

**WHAT THIS STUDY ADDS**

- Modelling results showed that existing LSFF programmes (vitamin A-fortified refined oil and iron and folic acid-fortified wheat flour) make substantial contributions to reducing inadequacy, but gaps remain that could be further closed by improving compliance with current standards, revising/expanding current standards and/or adding new fortified food vehicles such as rice, with careful consideration given to subnational populations and demographic groups where risk of high intakes may exist. In some regions, targeted micronutrient interventions, such as supplementation, may also be needed.

**HOW THIS STUDY MIGHT AFFECT RESEARCH, PRACTICE OR POLICY**

- These results provide evidence-based information that can help inform policy discussions around developing safe and effective LSFF programmes and identifying strategies to improve compliance with standards as part of a coordinated set of micronutrient intervention programmes in Senegal.

## Introduction

 Recent global estimates suggest that, worldwide, micronutrient deficiencies afflict over half of preschool-aged children and two-thirds of women of reproductive age (WRA),^1^ the consequences of which include increased risk of morbidity and mortality, poor growth, impaired cognitive function and reduced work capacity, which generate significant private and social costs.^2^,^5^ The most recent national biomarker data collected in Senegal (2010) showed that 12.8% of children 12–59 months of age were deficient in vitamin A^6^ and half of children were deficient in zinc.^7^ Among women, the same micronutrient survey data indicated that the prevalence of vitamin A deficiency (based on plasma retinol and using a cut-off of 0.70 µmol/L) was 2.4%, 59% of WRA were zinc deficient,^7^ and 54.8% were deficient in folate.^8^

Large-scale food fortification (LSFF) can play an important role in improving the micronutrient adequacy of diets and reducing the burden of deficiency.^3 9^ Here, we focus on micronutrient inadequacy, defined as intake of micronutrients that is inadequate to meet theoretical requirements to fulfil metabolic and functional roles.^10^ Micronutrient deficiency, assessed by valid biomarkers for tissue reserves or nutrient-dependent function, may result from inadequate intake and/or other factors, including poor micronutrient absorption and bioavailability, or altered metabolism; however, inadequate intake is a primary cause of deficiency.^2^ Senegal currently mandates the fortification of refined oil with vitamin A and the fortification of wheat flour with iron and folic acid. Previous work has established that some of the conditions for successful and impactful LSFF programmes are already in place in Senegal, including high reach of staple foods^11 12^ and government engagement.^13^ A recent study assessed the potential contributions of adding micronutrient-fortified bouillon to Senegal’s current LSFF programme.^14^ However, to our knowledge, an assessment focused specifically on understanding the contributions of the existing LSFF programme (including expanding the current programme to include additional micronutrients in fortified wheat flour) has not been conducted. Also, given the widespread consumption of rice in Senegal, there has been interest in the possibility of rice fortification,^12 15^ but a formal analysis of the potential for rice fortification to help fill micronutrient gaps in Senegal has, to our knowledge, not been conducted.

To fill these gaps in evidence, the first objective of this study was to establish ‘baseline’ dietary micronutrient needs in Senegal via an assessment of the prevalence of dietary inadequacies of vitamin A, thiamin (vitamin B_1_), riboflavin (vitamin B_2_), niacin (vitamin B_3_), folate (vitamin B_9_), vitamin B_12_, iron and zinc among WRA (15–49 years) and young children (aged 6–59 months) without the contribution of LSFF or other micronutrient interventions. The second objective was to model the contributions of the existing refined oil and wheat flour fortification programmes in Senegal to meeting the micronutrient requirements of WRA and children, including exploring how investments in improving compliance with the existing standards might improve these contributions. As described below, Senegal’s current wheat flour fortification standard mandates wheat flour be fortified with iron and folic acid only, and the mandatory fortification level for folic acid is below the current WHO guidelines.^16^ Therefore, another aim of the study was to assess the potential contributions of revising the wheat flour fortification standard to align with current WHO recommendations and to include additional micronutrients in the wheat flour standard if they are inadequate in baseline diets. Finally, given evidence on the potential for rice fortification in Senegal,^12^ a final aim was to assess the potential contributions of a mandatory national standard for rice fortification in Senegal. When assessing the contribution of LSFF programmes and, especially, when considering revised and/or new programmes, it is important to also assess any associated risks. To that end, we also estimated the risk of high micronutrient intakes associated with individual and combined LSFF programmes.

We estimated baseline micronutrient adequacy and modelled the contributions of LSFF at the national level but also subnationally by urban and rural residence and by region of residence. The subnational analyses were conducted to help identify where LSFF is, or could be, adequate to meet the micronutrient requirements of most of the population and also where additional, potentially targeted interventions might be needed to complement LSFF. We focused the analyses on WRA and children given their high micronutrient requirements relative to other population groups.^2^ Also, because children 6–59 months of age are a target population for high-dose vitamin A supplementation (VAS) in Senegal, it is important to assess micronutrient adequacy and examine risk for high intake among this group.

This modelling work was done as a part of USAID’s Feed the Future flagship fortification project, Advancing Opportunities for Food Fortification to Reinforce Diets (USAID AFFORD). The project seeks to safely and sustainably reduce micronutrient inadequacies and improve diets, particularly for women and children, through LSFF of staple foods. From January to June 2023, USAID AFFORD conducted three assessments in Senegal, including a dietary needs and economic modelling assessment (the dietary needs findings make up much of this paper), a policy enabling environment assessment, and an industry assessment, in close collaboration with the government of Senegal (specifically the National Nutrition Development Council) and the USAID Senegal mission.

## Methods

### Baseline prevalence of micronutrient inadequacy

We estimated the baseline prevalence (ie, without the contributions of LSFF or other micronutrient interventions) of apparent micronutrient inadequacies using household-level food consumption data from the 2018–2019 Enquête Harmonisée sur les Conditions de Vie des Ménages (EHCVM).^17^ The 2018–2019 EHCVM, which was implemented by the National Agency of Statistics and Demography in Senegal, is a household consumption and expenditure survey that collected data on household living conditions from 7156 households and is representative at the national level, by region, and by urban and rural residence. We requested and received approval from ANSD to use the 2018–2019 EHCVM data, which we used in compliance with ANSD’s data access policy. The dataset is now publicly available from the World Bank Microdata Library.^18^ All estimates account for survey weights. Note that we refer to ‘apparent’ food consumption and ‘apparent’ micronutrient adequacy as a reminder that these estimates are based on household-level data, which require imposing a number of assumptions (described below) to generate individual-level estimates.

The survey included a food consumption module in which the household respondent was asked to recall the quantity of each of 140 prespecified food items (see online supplemental table S1 for the list of food items) consumed by household members during the 7 days preceding the survey (food consumption module data were available for 7129 households). Using conversion factors provided with the survey data, we converted all reported quantities of food consumed to grams and calculated the average daily apparent quantities consumed. We adjusted for extreme outliers in reported quantities of food consumed by identifying the food-specific and region-specific 95th percentile of daily apparent household consumption per adult male equivalent (AME) (ie, we normalised the total quantity consumed across households by dividing that quantity by the number of AMEs in the households) and replacing quantities above the 95th percentile with the value at the 95th percentile.

Next, we estimated the energy and micronutrient contents of each food in the food list by matching it with a food composition table (FCT) entry. Matches were made based on input from in-country collaborators. Aggregate or generic food items were matched with several FCT entries to estimate the nutrient contents based on an average or weighted average (eg, the food item ‘sweet potato’ was matched with white-flesh sweet potato (assigned 80% wt) and orange-flesh sweet potato (assigned 20% wt)). Almost all matches were made with food items in the 2019 West African FCT,^19^ supplemented with entries from the Nutrition Coordinating Center Nutrient Database for Standard Reference^20^ and the Malawian FCT^21^ for several foods that did not have appropriate matches in the West African FCT. Where applicable, we adjusted the total quantity of food apparently consumed by the household for the edible portion and yield factor from cooking. We did not adjust for food waste.

We assessed the micronutrient adequacy of household diets for meeting the needs of WRA and children 6–59 months of age based on the nutrient density of the household diet. For each micronutrient (vitamin A, thiamin, riboflavin, niacin, folate, vitamin B_12_, iron and zinc), we calculated the nutrient density of the household diet as the ratio of total daily apparent intake of the micronutrient to total daily energy intake, expressed per 1000 kcal by multiplying that ratio by 1000. Except for iron, we assessed the adequacy of the household diet for meeting the needs of WRA and children using the cut-point method. Specifically, we compared the nutrient density of the household diet to the critical nutrient density of a randomly selected WRA and/or child aged 6–59 months in the household, where critical nutrient densities were calculated as the age-specific and sex-specific estimated average requirement (EAR) divided by the age-specific and sex-specific energy requirements of the WRA or child, expressed per 1000 kcal. Because the iron requirements of children and non-pregnant WRA are not normally distributed, we used the full probability method, based on the iron density of diets, to asses iron adequacy.^3^ In short, estimating iron adequacy based on the full probability method accounts for skewed iron requirements by comparing iron intakes (or, in this case, densities) to a distribution of requirements to calculate the probability of inadequacy.

EARs for all micronutrients except zinc were from the US Institute of Medicine^22^ (online supplemental table S2). Based on dietary patterns in Senegal, we assumed a 10% bioavailability of iron.^3^ Zinc adequacy was assessed relative to physiological zinc requirements for WRA^23^ and children.^24^ Using published algorithms for children^25^ and adults^26^ to estimate absorbable zinc, for each target household member, we calculated fractional zinc absorption as the ratio of absorbed zinc to total zinc. Then, we estimated dietary zinc requirements (and critical nutrient densities) by adjusting physiological zinc requirements by the estimated person-specific fractional zinc absorption. Note that we were not able to assess iodine adequacy because the West African FCT does not include estimates of the iodine content of foods.

Pregnancy status was not collected in the EHCVM survey. Therefore, we assessed micronutrient adequacy among pregnant and non-pregnant WRA by first assuming all WRA in the sample were pregnant and estimating the prevalence of inadequacy and then assuming all WRA in the sample were not pregnant and estimating the prevalence of inadequacy. These separate estimates were then combined into an overall estimate of micronutrient adequacy among pregnant and non-pregnant WRA as a weighted average of the two estimates, where weights were based on 2019 Demographic and Health Survey (DHS) estimates of the proportion of WRA currently pregnant and not pregnant.^27^

### Consumption of fortifiable foods

We estimated the apparent consumption of select fortifiable foods (refined oil, wheat flour and imported rice) for WRA and children using the AME method (as explained below, only imported rice, which was listed separately from local rice in the EHCVM food list, was considered fortifiable in this analysis). The AME method is based on the assumption that the intrahousehold distribution of food is in proportion to each member’s age-specific and sex-specific energy requirements.^28^ Based on age and sex information collected in the household roster, we assigned an AME weight to each household member, calculated as the ratio of each household member’s assumed energy requirement to the energy requirement of a male aged 18–30 years (online supplemental file 1).^29^ Then, each household member was assigned an AME ratio, calculated as his/her AME weight divided by the total number of AMEs in the household. Apparent consumption of each fortifiable food vehicle (including consumption of processed products containing the food vehicle using fortifiable food equivalents) was then calculated as total household apparent consumption of food vehicle multiplied by the AME ratio of the selected WRA and/or child in the household.

### Modelled contribution of LSFF

To assess the contribution of LSFF to meeting the micronutrient requirements of WRA and children, we modelled a set of scenarios based on Senegal’s existing refined oil (vitamin A, 17.5 mg/kg) and wheat flour (iron, 60 mg/kg and folic acid, 2.5 mg/kg) fortification standards (online supplemental table S3). We also modelled hypothetical scenarios to assess the potential contribution of (1) revising the mandated folic acid fortification level from 2.5 mg/kg to 5 mg/kg to align with current WHO guidelines^16^ and (2) adding thiamin (3 mg/kg), riboflavin (2 mg/kg), vitamin B_12_ (0.02 mg/kg) and zinc 55 (mg/kg) to the wheat flour fortification standards at fortification levels in alignment with current WHO guidance. Finally, we modelled the hypothetical establishment of a rice fortification standard based on the current World Food Programme’s technical specifications for rice fortification^30^ (online supplemental table S3). Note that we converted folic acid from fortification to dietary folate equivalents to account for the higher bioavailability of folic acid compared with dietary folate.^31^

Informed by recent qualitative and quantitative testing of market samples of refined oil and wheat flour in Senegal, we modelled refined oil fortification under a status quo current compliance scenario (59% of fortifiable refined oil fortified to an average of 82% of the national standard^32^) and an improved compliance scenario assuming that 75% of refined oil was fortified to 100% of the standard (online supplemental table S4). We also modelled a status quo current compliance scenario for wheat flour (76% of wheat flour fortified to an average of 43% of the national standard^32^) as well as an improved compliance scenario in which we assumed 76% of wheat flour was fortified to 100% of the standard. In line with the improved compliance scenarios, we modelled the potential contribution of rice fortification assuming 75% of fortifiable rice was fortified to the specified standard. Note that, currently, ~60% of Senegal’s rice supply is imported and could potentially be fortified prior to importation.^13^ In our modelling, we assumed all imported rice was fortifiable. A negligible share of the rice supply (~1.5%) is domestically refined at industrial scale (and hence may be feasible for fortification). We did not account for domestically milled rice in the modelling. Note that all modelling assumptions we applied at the national level.

For each scenario, we increased the nutrient content of the food vehicle to account for additions via LSFF, recalculated the nutrient density of the household diet and then compared the recalculated nutrient density of the household diet to critical nutrient densities for WRA and children. Where relevant, assumed fortification levels at the point of consumption were based on adjustments for expected micronutrient losses between the point of fortification and the point of consumption (online supplemental table S5).

For each scenario and micronutrient for which a tolerable upper intake level (UL) for safe consumption exists (vitamin A in the form of preformed retinol, folic acid (excluding dietary folate), iron and zinc), we also estimated the risk of high intake by comparing the nutrient density of the household diet to the critical upper density for the micronutrient. UL values were taken from the US Institute of Medicine^22 33^ (online supplemental table S6).

### Accounting for VAS

Senegal implements a national VAS programme in which high-dose vitamin A supplements are provided every 6 months to children aged 6–59 months. Given the importance of balancing micronutrient contributions from fortification and supplementation where the risk of high intake may be a concern, we also modelled the contribution of VAS to meeting micronutrient requirements as well as the proportion of children who receive VAS (or do not receive VAS) and (1) are at risk of high retinol intake with LSFF, (2) have adequate (but not high) vitamin A intake with LSFF and (3) have inadequate vitamin A intake with LSFF. Receipt of VAS was based on 2019 DHS coverage estimates and linked to the ECHVM data (see online supplemental methods).^27^ Following Engle-Stone *et al*,^34^ receipt of VAS was converted to a daily equivalent intake of 167 µg RAE/day. This daily equivalent value was incorporated into the vitamin A density of the diet and compared with the vitamin A requirements of children aged 6–59 months.

### Patient and public involvement

Because these were analyses of secondary data, patients and public were not involved in the design, conduct or reporting of this research. Results were disseminated at a validation workshop in Senegal.

## Results

### Prevalence of baseline micronutrient inadequacies

Based on the nutrient density of household diets, the national baseline (ie, without accounting for the contributions of LSFF or other micronutrient interventions) prevalence of apparent micronutrient inadequacy among WRA was above 60% for all micronutrients analysed except for niacin and vitamin B_12_ and above 70% for all micronutrients except niacin, vitamin B_12_ and iron among children (table 1). There was notable subnational variation in the baseline prevalence of inadequacy by region. Among WRA, there were particularly large discrepancies in the prevalence of apparent vitamin A inadequacy (ranging from 52% in the Ziguinchor Region to 91% in the Saint-Louis and Louga regions), vitamin B_12_ (14% in the Diourbel Region to 80% in the Kedougou Region) and zinc (44% in the Ziguinchor Region to 82% in the Saint-Louis Region). The largest regional discrepancies in the prevalence of apparent inadequacy among children were for vitamin A (ranging from 5% in the Ziguinchor Region to 90% in the Saint-Louis Region), folate (40% in the Kaffrine Region to 95% in the Saint-Louis Region) and vitamin B_12_ (9% in the Diourbel Region to 76% in the Kedougou Region). Given the very low prevalence of apparent niacin inadequacy nationally and subnationally among both WRA and children, we did not model the potential addition of niacin to wheat flour.

**Table 1:** Prevalence of apparent micronutrient inadequacy without LSFF among women of reproductive age and children based on the 2018–2019 Enquête Harmonisée sur les Conditions de Vie des Ménages survey

	Prevalence of apparent inadequacy, % (95% CI)							
	Vitamin A	Thiamin	Riboflavin	Niacin	Folate	Vitamin B_12_	Iron*	Zinc
Women of reproductive age								
Senegal	70 (69, 72)	92 (91, 93)	95 (94, 96)	1 (1, 1)	92 (91, 92)	29 (28, 31)	61	66 (65, 68)
By residence								
Urban	59 (57, 62)	95 (94, 96)	91 (89, 92)	1 (0, 1)	97 (97, 98)	20 (18, 22)	63	59 (57, 62)
Rural	82 (80, 83)	88 (87, 90)	99 (98, 99)	1 (1, 2)	86 (84, 87)	40 (38, 42)	58	74 (72, 76)
By region								
Dakar	54 (50, 57)	96 (94, 97)	86 (83, 89)	1 (0, 1)	98 (97, 99)	16 (13, 19)	67	52 (48, 56)
Ziguinchor	52 (47, 57)	98 (96, 99)	97 (96, 99)	1 (0, 1)	98 (97, 100)	54 (48, 59)	67	44 (39, 49)
Diourbel	84 (81, 88)	94 (91, 96)	100 (100, 100)	0 (0, 1)	89 (86, 93)	14 (10, 18)	61	73 (68, 77)
Saint-Louis	91 (88, 94)	99 (98, 100)	98 (97, 99)	3 (1, 4)	99 (90, 91)	34 (29, 38)	80	82 (78, 86)
Tambacounda	78 (74, 82)	82 (78, 86)	100 (99, 100)	2 (0, 4)	85 (81, 89)	68 (64, 73)	56	71 (66, 76)
Kaolack	67 (62, 71)	88 (85, 91)	98 (97, 99)	0 (0, 1)	83 (80, 87)	27 (23, 31)	54	72 (68, 77)
Thies	77 (73, 81)	94 (92, 96)	97 (95, 98)	1 (0, 1)	94 (92, 96)	14 (11, 17)	64	74 (70, 78)
Louga	91 (88, 94)	93 (89, 96)	98 (97, 99)	1 (0, 3)	93 (83, 88)	26 (21, 30)	69	80 (75, 84)
Fatick	74 (69, 79)	84 (79, 88)	99 (98, 100)	0 (0, 1)	74 (69, 78)	35 (29, 40)	51	69 (64, 75)
Kolda	54 (49, 59)	85 (81, 89)	96 (94, 98)	1 (0, 1)	88 (85, 92)	66 (62, 71)	53	61 (56, 66)
Matam	88 (84, 91)	89 (86, 92)	97 (95, 99)	4 (1, 7)	97 (95, 99)	40 (34, 46)	62	80 (75, 85)
Kaffrine	79 (75, 83)	79 (74, 83)	99 (98, 100)	0 (0, 1)	70 (65, 75)	40 (35, 46)	51	79 (75, 83)
Kedougou	62 (56, 67)	77 (72, 81)	98 (96, 99)	0 (0, 1)	91 (88, 94)	80 (76, 85)	55	62 (56, 67)
Sedhiou	62 (56, 67)	96 (94, 98)	98 (96, 99)	1 (-1, 2)	90 (87, 94)	69 (64, 74)	58	68 (63, 74)
Children 6–59 months								
Senegal	70 (68, 72)	86 (85, 88)	94 (93, 95)	2 (2, 3)	76 (75, 78)	25 (23, 26)	43	74 (73, 76)
By residence								
Urban	58 (55, 61)	92 (91, 94)	89 (88, 91)	2 (1, 3)	87 (85, 89)	14 (12, 15)	46	75 (72, 78)
Rural	80 (79, 82)	82 (80, 83)	98 (97, 98)	3 (2, 3)	68 (66, 70)	34 (32, 36)	40	74 (72, 76)
By region								
Dakar	53 (48, 59)	94 (92, 97)	84 (81, 88)	2 (1, 3)	91 (87, 94)	10 (7, 13)	50	73 (68, 78)
Ziguinchor	50 (44, 57)	96 (93, 98)	95 (92, 98)	0 (0, 0)	88 (84, 92)	39 (32, 45)	47	59 (52, 65)
Diourbel	80 (76, 85)	89 (85, 93)	98 (96, 100)	1 (0, 2)	73 (68, 78)	9 (5, 12)	44	78 (74, 83)
Saint-Louis	90 (86, 93)	97 (95, 99)	98 (96, 99)	7 (4, 10)	95 (92, 97)	26 (21, 31)	59	89 (85, 92)
Tambacounda	78 (74, 83)	74 (69, 79)	99 (99, 100)	5 (3, 8)	68 (62, 73)	61 (55, 67)	38	70 (64, 75)
Kaolack	66 (61, 71)	78 (74, 82)	96 (94, 98)	2 (1, 3)	55 (50, 61)	21 (16, 25)	38	71 (66, 75)
Thies	76 (71, 80)	88 (84, 91)	95 (93, 97)	3 (1, 5)	80 (76, 85)	13 (10, 17)	46	80 (75, 84)
Louga	88 (85, 92)	88 (84, 92)	98 (96, 99)	3 (1, 4)	83 (78, 87)	20 (15, 25)	51	83 (78, 88)
Fatick	76 (70, 81)	78 (72, 84)	99 (98, 100)	1 (0, 2)	44 (38, 51)	30 (24, 36)	35	64 (57, 70)
Kolda	52 (46, 58)	77 (72, 82)	95 (92, 97)	0 (0, 0)	70 (65, 76)	56 (50, 62)	35	63 (57, 69)
Matam	87 (83, 91)	80 (75, 85)	93 (90, 96)	5 (2, 8)	86 (81, 90)	33 (27, 40)	45	87 (83, 91)
Kaffrine	82 (77, 86)	67 (61, 73)	98 (96, 100)	1 (0, 1)	40 (35, 46)	40 (34, 46)	33	72 (66, 77)
Kedougou	57 (50, 63)	69 (63, 75)	96 (93, 99)	0 (0, 0)	76 (71, 82)	76 (70, 81)	39	66 (60, 72)
Sedhiou	58 (52, 64)	93 (90, 97)	96 (94, 98)	1 (0, 3)	73 (67, 78)	56 (50, 63)	39	68 (62, 74)

* Estimation of confidence intervalCIs was not was not possible for iron given the methods used to estimate iron inadequacy.

LSFFlarge-scale food fortification

### Apparent consumption of refined oil, wheat flour and imported rice

Most households in Senegal reported consuming purchased refined oil or products containing refined oil (90% of households) and wheat flour or products containing wheat flour (89% of households) in any amount in the 7 days preceding the survey (table 2). Across regions and households residing in urban and rural areas, household consumption of these food vehicles was reported by at least 70% of households. Consumption of imported rice in the 7 days preceding the survey was reported by 52% of households nationally, with similar proportions of urban (55%) and rural (49%) of households reporting imported rice consumption. Across regions, imported rice consumption ranged from 12% in the Saint-Louis Region to 78% in the Fatick Region.

**Table 2:** Apparent consumption of refined oil, wheat flour and imported rice based on the 2018–2019 Enquête Harmonisée sur les Conditions de Vie des Ménages survey

	Households consuming* (%)			Median apparent consumption among consumers, grams/day		
	Refined oil	Wheat flour	Imported rice	Refined oil	Wheat flour	Imported rice
				WRA; children†	WRA; children†	WRA; children†
Senegal	90	89	52	45; 21	55; 26	200; 99
By residence						
Urban	95	94	55	48; 23	58; 28	192; 96
Rural	85	84	49	40; 19	49; 23	209; 103
By region						
Dakar	94	96	59	48; 24	60; 28	180; 91
Ziguinchor	88	77	59	24; 11	44; 21	219; 111
Diourbel	74	97	34	48; 23	59; 29	208; 99
Saint-Louis	97	94	12	64; 33	56; 28	188; 84
Tambacounda	86	70	56	29; 14	35; 14	210; 104
Kaolack	91	85	77	39; 18	44; 21	191; 94
Thies	94	95	35	51; 24	56; 27	192; 89
Louga	94	97	44	60; 30	52; 24	231; 116
Fatick	92	83	78	35; 16	39; 19	182; 89
Kolda	81	71	64	15; 7	30; 14	182; 121
Matam	98	88	44	57; 29	68; 33	243; 117
Kaffrine	82	81	66	38; 16	46; 20	193; 92
Kedougou	85	70	52	20; 9	33; 14	225; 109
Sedhiou	93	62	73	22; 11	43; 22	267; 130

* Households reporting any consumption of the food vehicle (or processed food containing the food vehicle), from household purchases, during the recall period categorizedcategorised as consuming the food vehicle.

† Children 6–59 months of age.

WRA, women of reproductive age;

Based on the subsamples of households that reported consuming these food vehicles in any amount, median apparent consumption of refined oil was 45 g/day among WRA and 21 g/day among children. Median apparent consumption of wheat flour among consumers was 55 g/day among WRA and 26 g/day among children, while median apparent imported rice consumption among consumers was 200 and 99 g per day among WRA and children, respectively. Subnationally, while there was little variation in quantities of each food vehicle apparently consumed between urban and rural consumers, median daily apparent consumption of refined oil was over four times higher in some regions than others (with the highest and lowest median apparent consumption in the Saint-Louis and Kolda regions, respectively), and median daily apparent consumption of wheat flour was over twice as high in the Matam Region as the Kolda Region. Daily average apparent consumption of imported rice was more similar across regions than refined oil or wheat flour.

### Modelled contribution of LSFF to meeting micronutrient requirements and risk of high intake

**Figure 1:**
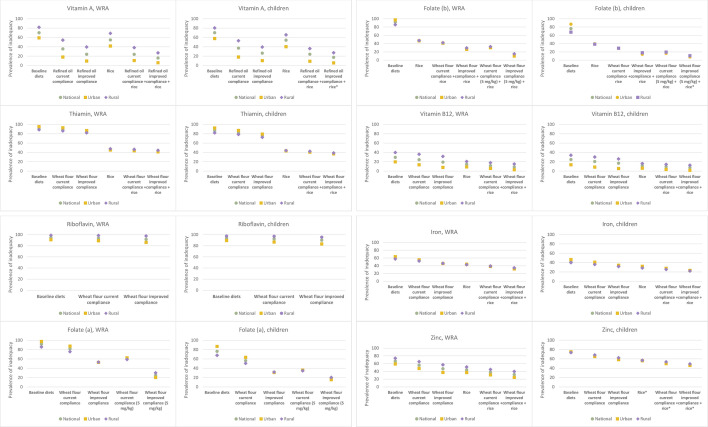
Prevalence of apparent micronutrient inadequacy among WRA and children without and with the contribution of micronutrients via LSFF under various modelling scenarios. Asterisks (*) indicate risk of high intake associated with the specific scenario at or above 5% nationally or in urban or rural subpopulations. Apparent risk of high vitamin A (in the form of preform retinol) intake among children with refined oil at improved compliance+rice fortification: 5% among urban children. Apparent risk of high folic acid intake among children with wheat flour (5 mg/kg) at improved compliance+rice fortification: 7% nationally, 8% among urban children and 6% among rural children. Apparent risk of high zinc intake (1) among children with (1) rice fortification: 32% nationally, 26% among urban children and 36% among rural children; (2) wheat flour at current compliance+rice fortification: 40% nationally, 37% among urban children and 43% among rural children and (3) wheat flour at improved compliance+rice fortification: 53% nationally, 52% among urban children and 54% among rural children. LSFF, large-scale food fortification; WRA, women of reproductive age.

The predicted contribution of existing and hypothetical LSFF programmes to the adequacy of household diets for meeting the micronutrient requirements of WRA and children aged 6–59 months is shown in figure 1. All point estimates at the national level, by urban and rural residence, and by region are available in online supplemental table S7–S19).

**Figure 2:**
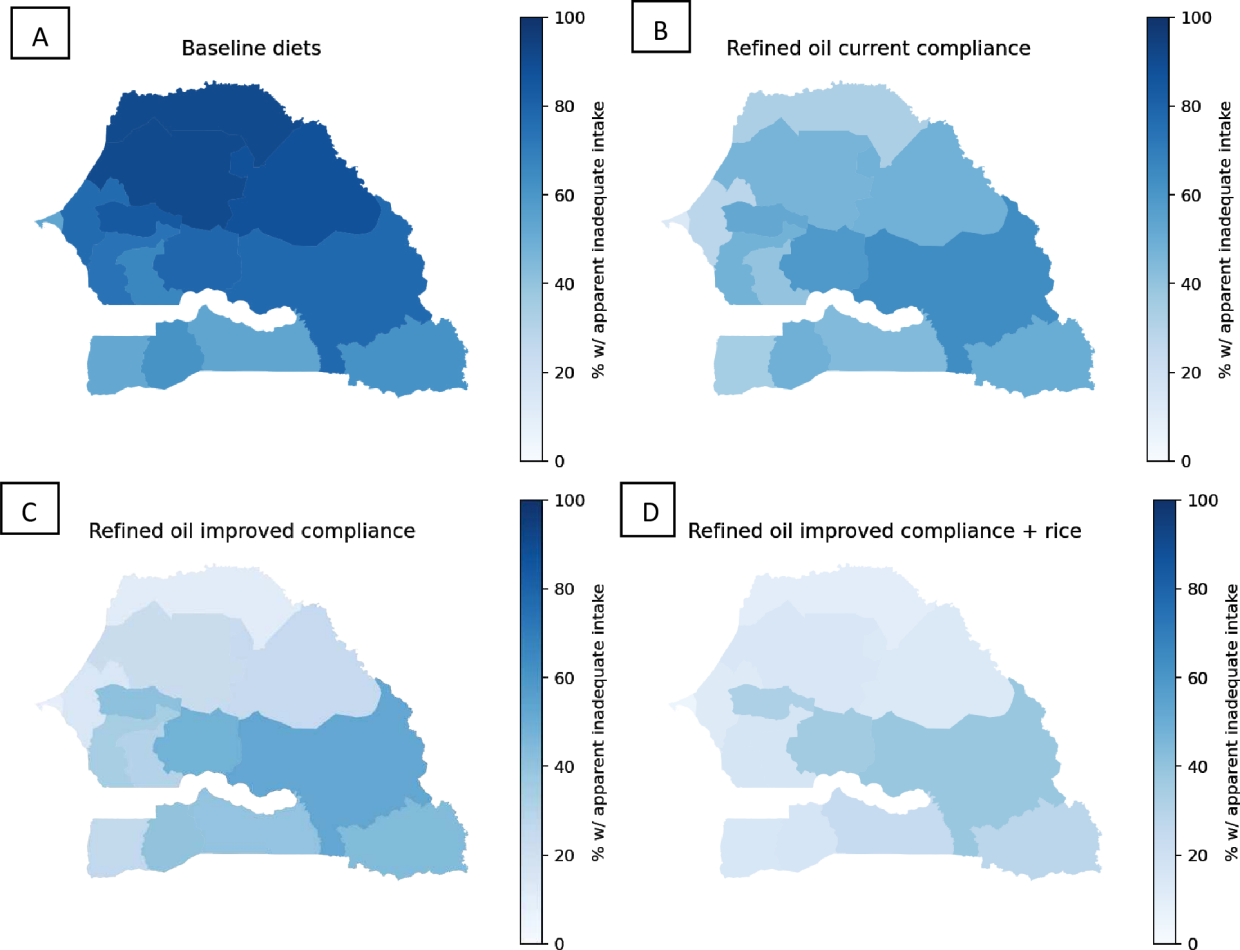
Maps of apparent vitamin A inadequacy among WRA without LSFF (A), with oil fortification at status quo compliance (B), with oil fortification at improved compliance (C) and with oil fortification at improved compliance plus rice fortification (D). LSFF, large-scale food fortification; WRA, women of reproductive age.

Vitamin A-fortified refined oil at current levels of estimated compliance with the national standard was predicted to reduce the prevalence of apparent inadequacy among both WRA and child by >30 percentage points (from 70% to 35% among WRA and from 70% to 37% among children), although inadequacy in rural areas remained above 50% among both population groups (figure 1; online supplemental table S7). If compliance with the refined oil fortification standard improved, the prevalence of vitamin A inadequacy was predicted to further decline to 24% among WRA and to 26% among children. Adding fortified rice to the refined oil fortification programme at current compliance was predicted to reduce the national prevalence of vitamin A inadequacy to 24% among both WRA and children. With the combination of fortified refined oil at improved compliance and rice fortification, the national prevalence of vitamin A inadequacy was predicted to decline to 16% and 17% among WRA and children, respectively, but even with this combination, the prevalence of inadequacy was >25% in rural areas. Across the regions of Senegal, the predicted prevalence of vitamin A inadequacy among WRA declined to between 14% in the Dakar Region and 64% in the Tambacounda Region with refined oil at current compliance, to between 8% (Dakar Region) and 52% (Tambacounda Region) with improved compliance with the refined oil standard (figure 2). Across regions, the addition of vitamin A-fortified rice to refined oil fortification with improved compliance was predicted to reduce vitamin A inadequacy among WRA to between 5% and 39%.

Across all modelled scenarios, the predicted prevalence of high vitamin A intake was 1% or lower among WRA (online supplemental table S8). However, while the national predicted risk of high vitamin A intake among children was 2% or lower for all modelled scenarios, risk of high intake exceeded 5% in the Dakar Region in all scenarios with refined oil fortification and reached 9% with the combination of refined oil at improved compliance plus rice fortification (the risk of high intake among urban children was also 5% in this scenario). Across all modelled LSFF scenarios, 1% of children nationally were predicted to be both at risk of high vitamin A intake with LSFF and also received VAS, and a similar proportion was at risk of high vitamin A intake without receiving VAS (online supplemental table S10). A predicted 23%–35% of children with adequate (but not high) intake with LSFF also received VAS (22%–45% with adequate intake with LSFF did not receive VAS). Finally, 5%–18% of children were predicted to have inadequate intake with LSFF and also received VAS (12%–36% had inadequate intake and did not receive VAS). In the Dakar Region, between 2% and 4% of children across LSFF scenarios were predicted to be both at risk of high vitamin A intake and also received VAS.

If Senegal added thiamin to the national wheat flour fortification standard at the WHO recommended level (3 mg/kg), the national prevalence of apparent inadequacy was predicted to drop less than 5 percentage points among both WRA with thiamin-fortified wheat flour at current compliance. With improved compliance, the national prevalence of thiamin inadequacy was predicted to decline by 8–11 percentage points, although given high baseline prevalence of inadequacy, the prevalence of inadequacy, nationally and in both urban and rural areas, remained above 80% among WRA and above 70% among children (figure 1; online supplemental table S11). Thiamin-fortified rice at 9.7 mg/kg (the WFP specification) was predicted to reduce the national prevalence of thiamin inadequacy among WRA from 92% to 47% and among children from 86% to 44%, with similar reductions in urban and rural areas. The combinations of rice and wheat flour fortification at current and improved compliance were predicted to reduce the prevalence of inadequacy by several additional percentage points.

Adding riboflavin to Senegal’s wheat flour fortification standard at the WHO recommended level (2 mg/kg) reduced the predicted prevalence of apparent inadequacy by several percentage points among WRA and children; however, in both the current and improved wheat flour compliance scenarios, the diets of 92%–94% of WRA and 90%–92% of children remained inadequate in riboflavin (online supplemental table S12). Because riboflavin turns fortified rice kernels orange/yellow, which can negatively impact consumer acceptance, riboflavin is typically not recommended for rice fortification,^35^ and therefore, we did not model rice fortification with riboflavin.

Senegal’s wheat flour fortification programme at currently estimated compliance was predicted to reduce the national prevalence of folate inadequacy among WRA by 10 percentage points (from 92% to 82%) and improved compliance with the standard was predicted to bring inadequacy to 53%, although inadequacy was predicted to remain above 70% in some regions (figure 1, folate (a); online supplemental table S13). The existing programme at current and improved compliance was also predicted to reduce folate inadequacy among children by 20 percentage points (from 76% to 56%) and 45 percentage points (to 31%), respectively. If Senegal revised the wheat flour fortification standard from 2.5 mg/kg to 5 mg/kg folic acid to align with current WHO guidance, predicted folate inadequacy among WRA was predicted to decline to 61% at current compliance and 25% with improved compliance, although inadequacy among WRA in the Kedougou and Sedhiou regions remained above 50% even with improved compliance. At this higher fortification level, folate inadequacy among children was predicted to decline to 18%–35%, depending on assumed compliance with the standard. There was no predicted risk of high folic acid intake among either WRA or children in any of these scenarios (online supplemental table S14).

Compared with wheat flour fortification alone, the combination of wheat flour at the current standard plus folic acid-fortified rice resulted in substantial reductions in folate inadequacy among WRA, reducing by 40 percentage points (from 82% to 42%) under the current compliance scenario and 25 percentage point (from 53% to 28%) under improved compliance (figure 1, folate (b)). Among children, this combination was predicted to reduce folate inadequacy by 28 percentage points (from 56% to 29%) under current wheat flour compliance and by 15 percentage points (from 31% to 16%) under improved wheat flour compliance. If the wheat flour standard was increased to 5 mg folic acid per kg, the combination of fortified wheat flour and rice was predicted to reduce folate inadequacy among WRA to between 13% and 31% and among children to between 9% and 18%, depending on assumed compliance with the wheat flour fortification standard. In terms of risk of high folic acid intake, fortified rice, either alone or in combination with fortified wheat flour, would put <1% of WRA at risk of high intake nationally and up to 3% of WRA in several regions with the combination or fortified rice and wheat flour at the higher fortification level of 5 mg/kg. Between 2% and 7% of children, nationally, were at risk of high folic acid intake with the combination or fortified rice and wheat flour, with much higher proportions of children in some regions at risk of high intake (eg, between 8% and 24% in the Ziguinchor Region; see online supplemental table S14).

**Figure 3:**
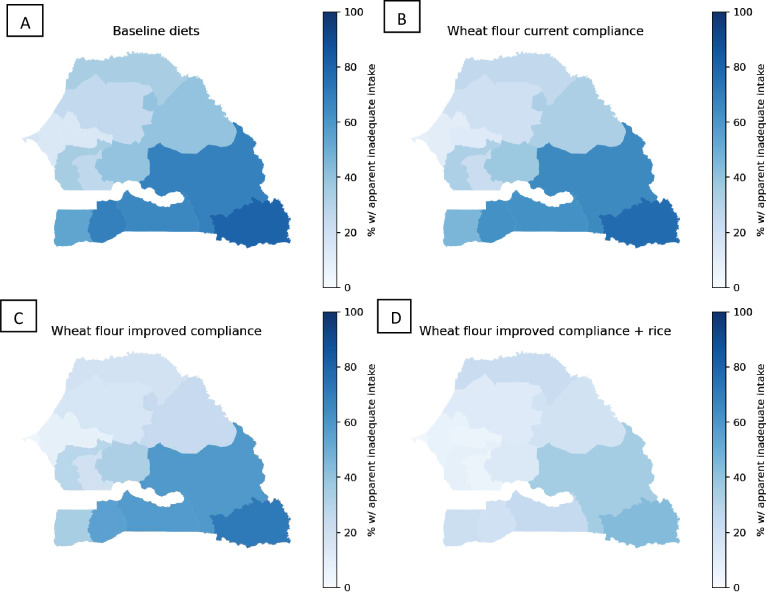
Maps of apparent vitamin B_12_ inadequacy among WRA without LSFF (A), with wheat flour fortification at current compliance (B), with wheat flour fortification at improved compliance (C) and with wheat flour fortification at improved compliance plus rice fortification (D). LSFF, large-scale food fortification; WRA, women of reproductive age.

Introducing vitamin B_12_ into the wheat flour fortification standard was predicted to reduce the national prevalence of apparent inadequacy among WRA from 29% to 24% (currently estimated compliance) or 19% (improved compliance) and from 25% to 21% (currently estimated compliance) or 17% (improved compliance) among children (figure 1; online supplemental table S15). However, with wheat flour fortification alone, the prevalence of vitamin B_12_ inadequacy was still >30% among WRA in rural areas (>25% among children) and remained above 50% among WRA in some regions (figure 3). Vitamin B_12_-fortified rice was predicted to reduce inadequacy to 15% nationally among WRA and to 12% nationally among children. The combination of fortified wheat flour and rice reduced inadequacy to 9%–12% nationally among WRA and to 8%–9% among children, although >30% of WRA would still have inadequate vitamin B_12_ in the Tambacounda Region as well as ~40% of both WRA and children in the Kedougou region.

The current wheat flour fortification programme was predicted to reduce apparent iron inadequacy among WRA by 7–15 percentage points (from 63% inadequate to 54% inadequate with current estimated compliance and to 46% inadequate with improved compliance) and by 5–10 percentage points among children (from 43% to 38% inadequate with current estimated compliance and to 33% with improved compliance), with reductions ranging from 8 to 21 percentage points among WRA and 7–15 percentage points among children across regions under the improved compliance scenario (figure 1; online supplemental table S16). The combination of iron-fortified rice and wheat flour at current or improved compliance was predicted to reduce inadequacy to 33%–39% and 23%–27% among WRA and children, respectively. There were no predicted risks of high iron intake among WRA or children as a result of wheat flour and/or rice fortification (online supplemental table S17).

Finally, adding zinc to Senegal’s wheat flour fortification standard (55 mg/kg) reduced the prevalence of apparent zinc inadequacy among WRA from 66% to between 47% and 56% nationally, depending on compliance with the standard, although the prevalence of inadequacy remained above 50% among WRA living in rural areas as well among women in many regions (figures1  4; online supplemental table S18). Among children, wheat flour fortification reduced zinc inadequacy from 74% to between 61% and 67% nationally. Rice fortified with zinc was predicted to reduce the national prevalence of zinc inadequacy by several percentage points more than wheat flour fortification, and the combination of wheat flour and rice fortification reduced the predicted prevalence of inadequacy to between 32% and 38% among WRA and 48% and 52% among children. However, rice fortification alone and in combination with wheat flour fortification resulted in a substantial proportion (33%–41%) of children at risk of high zinc intake nationally. With wheat flour fortification alone, the national risk of high zinc intake among children was <5% but reached 5%–6% in the Dakar, Kolda and Matam regions with improved compliance.

**Figure 4:**
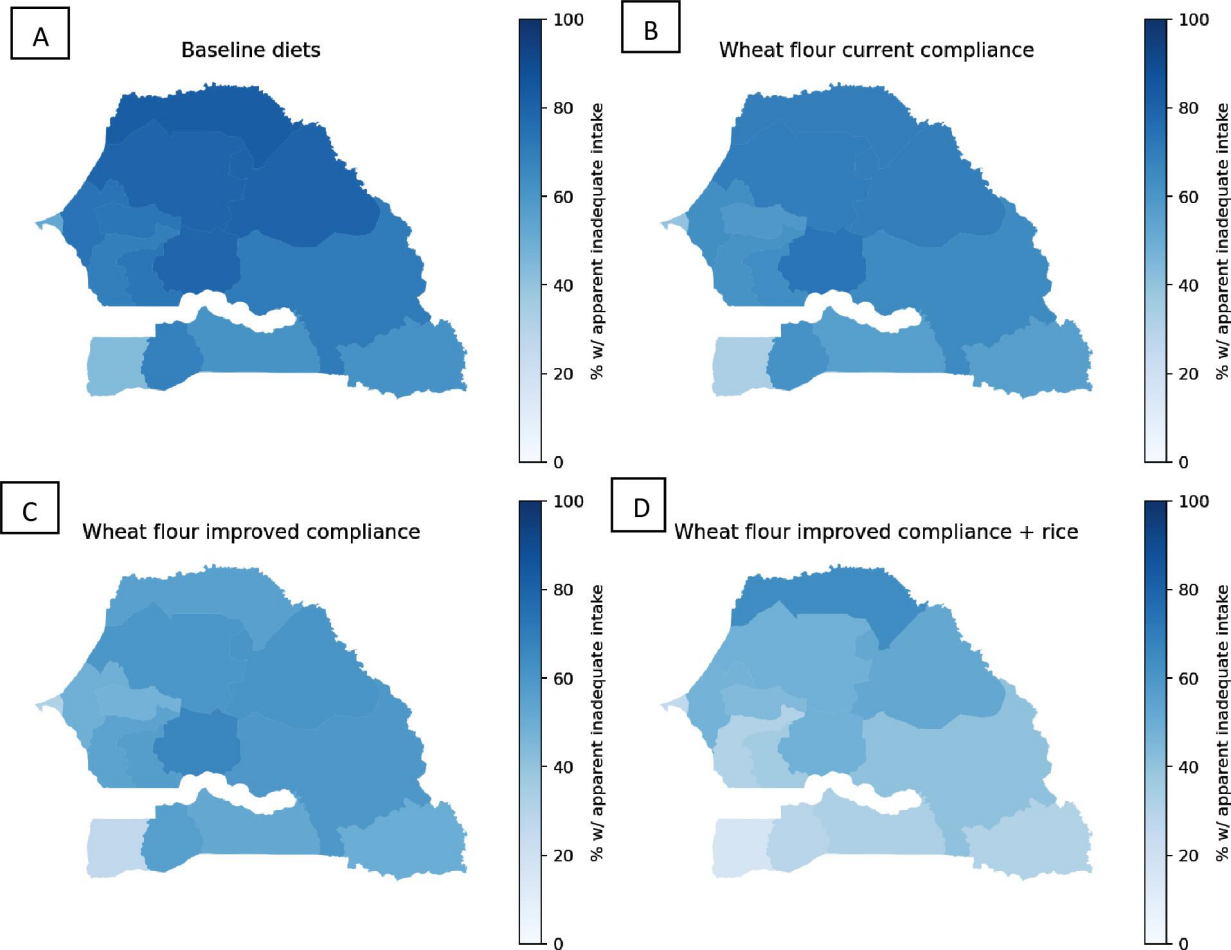
Maps of apparent zinc inadequacy among WRA without LSFF (panel A), with wheat flour fortification at current compliance (panel B), with wheat flour fortification at improved compliance (panel C), and with wheat flour fortification at improved compliance plus rice fortification (panel D). LSFF, large-scale food fortification; WRA, women of reproductive age.

## Discussion

In the long term, sustainable improvements in the micronutrient adequacy of diets should ideally stem from improvements in dietary diversity via adequate availability and consumption of nutrient-dense foods. LSFF can help fill dietary gaps now and as a complement to dietary diversification strategies going forward. Using household food consumption data, we found that, without fortification, a high proportion of household diets in Senegal were inadequate to meet the micronutrient requirements of WRA and children. The estimated prevalence of apparent inadequacy exceeded 70% for vitamin A, thiamin, riboflavin, folate and zinc; apparent iron inadequacy was 61% among WRA (43% among children), and approximately a quarter of household diets provided inadequate vitamin B_12_.

Modelling results showed that Senegal’s current refined oil fortification programme is contributing to substantial reductions in vitamin A inadequacy, reducing the prevalence of inadequacy among both WRA and children by more than 40 percentage points, with reductions of an additional ~10 percentage points possible if factors behind low vitamin A content in fortified oil could be addressed (eg, enhanced regulatory monitoring and enforcement if oil processor compliance is poor). At the same time, the risk of high vitamin A intakes among children in this improved compliance scenario was estimated to be 2% nationally, 4% among urban children and 8% among children in the Dakar Region. In this scenario, the predicted overlap in children with vitamin A intake above the UL and receipt of high-dose VAS was 1% of children nationally, 2% in urban areas and 4% in the Dakar region. In addition, 33% of children with adequate, but not high, vitamin A intake in this scenario also received VAS; any potential health risks associated with this combination are unclear. Thus, the benefits of reducing inadequacy must be weighed against the possibility of high intakes, and if there are concerns that vitamin A intakes among children may be too high at mandated fortification levels, reconsidering the refined oil fortification standard and/or developing a targeted (vs national) VAS programme may be warranted.

Senegal’s existing wheat flour fortification programme was predicted to make substantial contributions to reducing folate inadequacy among WRA and children, especially if compliance with the standard is improved (reducing folate inadequacy to 53% among WRA and to 31% among children). In a scenario in which the wheat flour fortification standard was revised to align with current WHO guidance (5 mg/kg folic acid instead of 2 mg/kg), the predicted prevalence of apparent folate inadequacy with improved compliance dropped to 25% among WRA and 18% among children nationally. However, inadequacy among WRA remained above 50% in several regions, potentially suggesting the need for fortification of additional food vehicles or other targeted intervention strategies. With existing wheat flour fortification, iron inadequacy among WRA remained around 50%, even with improved compliance, again suggesting the potential need for iron fortification of additional food vehicles and/or other strategies to help meet iron requirements.

Expanding Senegal’s existing wheat flour fortification programme to include B vitamins and zinc could help meet requirements, but, based on dietary gaps and wheat flour consumption patterns, the predicted impacts on the prevalence of inadequacy varied. In particular, potential reductions in thiamin and riboflavin inadequacy via wheat flour fortification (at levels consistent with WHO guidance, which are intended to replace losses in these micronutrients during milling^16^) were modest; higher fortification levels could be considered to help meet dietary requirements. Introducing vitamin B_12_ into the wheat flour fortification standard was predicted to reduce the national prevalence of inadequacy to below 20% under the improved compliance scenario, although diets were still inadequate for >50% of WRA and children in some regions. Zinc-fortified wheat flour reduced the prevalence of zinc inadequacy by ~20 percentage points among WRA (~10 percentage points among children), although 47% of WRA and 61% of children remained inadequate.

Given these findings—that even with an expanded wheat flour fortification programme modelled at improved compliance, the national predicted prevalence of dietary micronutrient inadequacies among one or both target populations remained near or exceeded 50% for thiamin, riboflavin, iron and zinc and remained above 50% in some regions for vitamin A, folate and vitamin B_12_—fortification of additional food vehicles may be needed to help fill remaining gaps. Our modelling results showed that the addition of a programme to import fortified rice could potentially have a large (ie, >10 percentage point) additional impact on reductions in inadequacy for most micronutrients. However, the addition of vitamin A, folic acid and/or zinc to fortified rice would require careful planning to avoid excess intake among children (eg, re-examining fortification standards where foods are cofortified with the same nutrients as rice). Predicted rates of excess intake among children were particularly high for zinc. However, this result should be interpreted in the context of a recent call to reconsider the zinc ULs for children, amid concerns that the current ULs are set too low to feasibly design effective zinc intervention programmes in areas with high risk of zinc deficiency and because zinc ULs are set below commonly observed usual dietary zinc intake levels without any apparent adverse effects.^36^ As such, informed decisions around the design of LSFF programmes to deliver zinc in Senegal would need to weigh the current evidence on both risk of zinc inadequacy and the risk of high intakes.^37^

The potential contributions of bouillon fortified with vitamin A, folic acid, vitamin B_12_, iron and/or zinc have been assessed and are reported elsewhere.^14^ In short, that study found that bouillon is consumed by almost all households, and bouillon fortification could bring about substantial reductions in dietary inadequacy of vitamin A, folate, vitamin B_12_ and zinc, including among poor and rural populations. Future modelling could assess complementarities and overlaps in the combined contributions of rice and bouillon fortification in Senegal. This information, alongside other important considerations such as cost, affordability and industry readiness, could help Senegal determine which additional food vehicles (or combinations of food vehicles) might feasibly, effectively and cost-effectively help fill remaining dietary micronutrient gaps.

When nationally representative 24-hour dietary recall data are not available, the use of household dietary data can provide policy-relevant evidence on the micronutrient adequacy of diets and the contribution of LSFF and other strategies for meeting requirements.^38 39^ However, the use of household-level food consumption data has several important limitations that should be kept in mind when interpreting evidence based on the analyses of these data. First, the estimates of apparent food consumption and micronutrient adequacy rely on the assumption that food is distributed within the household in proportion to members’ age-specific and sex-specific energy requirements. Depending on the degree to which that assumption matches reality within households, using the household diet to assess adequacy of specific household members may be inaccurate, and if specific household members consume relatively more or less fortifiable foods than their relative energy requirements would suggest, the modelled impact of LSFF for meeting their requirements will likewise be inaccurate. Also, household food consumption is based on one household member’s recall of foods consumed from a set food list, and foods consumed away from home are commonly inadequately captured in these data. As a result, food consumption, potentially including processed food products containing wheat flour and/or refined oil, and micronutrient intake estimates are prone to recall and measurement error. We assessed adequacy based on the nutrient density of the household diet, which is a measure of the quality of the diet^40^ that can help address measurement or recall errors in reported quantities of foods consumed that are reflective of the typical household diet. However, these energy-adjusted measures cannot account for systematic under-reporting or over-reporting of foods that differ substantially in nutrient content from the typical household diet. Related, given inadequate accounting for foods consumed away from home, if processed foods containing wheat flour or refined oil are commonly consumed outside the home in Senegal, the estimated contribution of the fortification of these vehicles may be underestimated. Finally, there are challenges in accounting for high-dose VAS in addition to daily vitamin A intake among children, but in contexts where both VAS and vitamin A fortification programmes are in place, it is important to examine these programmes together. Beyond these limitations, we also modelled the potential addition of rice fortification to Senegal’s LSFF programmes based on WFP technical specifications, which were not designed specifically for Senegal. The development of rice fortification standards would ideally consider a variety of fortification levels for each micronutrient, alongside considerations around cost and technical feasibility.

## Conclusion

Taken together, these analyses showed substantial dietary micronutrient inadequacies among WRA and children in Senegal and the expected contributions of existing LSFF programmes to reducing inadequacies of vitamin A, iron and folic acid. Investments in strengthening the monitoring and enforcement system for refined oil and wheat flour could improve the impact of those programmes, and the wheat flour fortification programme could be revisited to revise fortification levels and/or mandate the inclusion of additional micronutrients. Given the widespread rice consumption throughout Senegal and the relatively high proportion (60%) of imported rice to the overall rice supply, adopting a national rice fortification standard could help reduce the prevalence of multiple micronutrient inadequacies; policy-makers in Senegal would need to consider how to approach the remaining ~40% of rice that is locally milled at small scale and how to design a rice fortification standard in the context of current efforts to increase and consolidate local rice production. Moreover, the design of any new programme (or redesign of existing programmes) should carefully consider potential risks of high intake among children. Under all modelled wheat flour fortification scenarios, the prevalence of riboflavin inadequacy remained above 85% nationally, suggesting the need for additional national strategies for meeting riboflavin requirements. For other micronutrients (vitamin A, iron and zinc), the prevalence of inadequacy remained above 25% in some regions, suggesting the potential need for targeted micronutrient interventions to fully close the micronutrient gap. When complemented with information on the cost and affordability of these intervention programmes, this evidence can help inform discussions and decisions around the design and implementation of a comprehensive set of safe and effective micronutrient interventions in Senegal.

## supplementary material

10.1136/bmjph-2024-001221 online supplemental file 1

## Data Availability

Data are available in a public, open access repository.
