# Supplementary material for: Current and potential contributions of large-scale food fortification to meeting micronutrient requirements in Senegal: a modelling study using household food consumption data
Source: BMJ Public Health. 2024 Nov 9;2(2):e001221. doi: 10.1136/bmjph-2024-001221 (PMC11737596; doi:10.1136/bmjph-2024-001221)
Supplement: online supplemental file 1 [file bmjph-2-2-s001.pdf]

Supplementary materials to "The current and potential contributions of large-scale food fortification to meeting micronutrient requirements in Senegal: A modeling study using Household Food Consumption Data"

Supplementary Methods

Linking EHCVM and DHS data

Consumption of micronutrient supplements was not collected in the Enquête Harmonisée sur les Conditions de Vie des Ménages (EHCVM) data. To estimate vitamin A adequacy among children 6-59 months of age who received a high-dose vitamin A supplement (VAS), we linked data on the receipt of VAS from the 2019 Demographic and Health Survey (DHS) with the EHCVM data. Specifically, using DHS data, we used a logit multivariate regression model to estimate the association between receipt of VAS in the six months preceding the survey and a set of child (child age and sex), household (household size, household wealth quintile, female-headed household, and education of head of household), and geographic characteristics (region of residence and urban/rural residence). We then used the estimated beta coefficients from the logit regression to predict the probability of receipt of VAS among children 6-59 months of age in the EHCVM data using Stata's "predict" postestimation command. By region, children with the highest probability of receipt of VAS were then assigned as receiving VAS until the proportion of children receiving VAS in the EHCVM data was equivalent to the region-specific prevalence in the DHS data.

Supplementary Tables

Supplemental Table S1. Food items included in the food consumption module of the 2018-2019 Enquête Harmonisée sur les Conditions de Vie des Ménages

Item code	Food item (French)	Food item (English)
1	Riz local brisé	Broken local rice
2	Riz local entier	Whole local rice
3	Riz importé brisé	Broken imported rice
4	Riz importé entier	Imported whole rice
5	Maïs en épi	Corn on the cob
6	Maïs en grain	Corn kernels
7	Mil	Mil
8	Sorgho	Sorghum
9	Blé	Wheat
10	Fonio	Fonio
11	Autres céréales	Other cereals
12	Farine de maïs	Corn flour
13	Farine de mil	Millet flour
14	Farine de blé local ou importé	Local or imported wheat flour
15	Autres farines de céréales	Other cereal flours
16	Pâtes alimentaires	Pasta
17	Pain moderne	Modern bread
18	Pain traditionnel	Traditional bread
19	Croissants	Croissants
20	Biscuits	Biscuits
21	Gâteaux	Cakes
22	Beignets, galettes	Donuts, pancakes
23	Viande de bœuf	Beef
24	Viande de chameau	Camel meat
25	Viande de mouton	Mutton
26	Viande de chèvre	Goat meat
27	Abats et tripes (foie, rognon, etc.)	Offal and tripe (liver, kidney, etc.)

28	Viande de porc	Pork meat
29	Poulet sur pied	Chicken on foot
30	Viande de poulet	Chicken meat
31	Viande d'autres volailles domestiques	Meat of other domestic poultry
32	Charcuterie (jambon, saucisson), conserves de viandes	Charcuterie (ham, sausage), canned meats
33	Gibiers	Game
34	Autres viandes n.d.a.	Other meat n.e.s.
35	Poisson frais yaboye ou obo (sardinelle)	Fresh yaboye or obo (sardinella) fish
36	Poisson frais thiof/ seudeu (baracouda)	Fresh fish thiof / seudeu (baracouda)
37	Poisson frais wass	Fresh fish wass
38	Autre Poisson frais (dorade, youfouf, rouget, siket [capitaine], thiarumbekh [mollette], …..)	Other Fresh fish (sea bream, youfouf, red mullet, siket [captain], thiarumbekh [mollette],… ..)
39	Poisson fumé Kethiakh (sardinelle)	Smoked fish Kethiakh (sardinella)
40	Autre Poisson fumé (Con fumé, yaboye ou obo fumé, …)	Other smoked fish (smoked con, smoked yaboye or obo, ...)
41	Poisson séché	Dried fish
42	Crabes, crevettes et autres fruits de mer	Crabs, shrimps and other seafood
43	Conserves de poisson	Canned fish
44	Lait frais	Fresh milk
45	Lait caillé, yaourt	Curdled milk, yogurt
46	Lait concentré sucré	Sweetened condensed milk
47	Lait concentré non-sucré	Unsweetened condensed milk
48	Lait en poudre	Powdered milk
49	Fromage	Cheese
50	Lait et farines pour bébé	Baby milk and flour
51	Autres produits laitiers	Other dairy products
52	Œufs	Eggs
53	Beurre	Butter
54	Beurre de karité	Shea Butter
55	Huile de palme rouge	Red palm oil
56	Huile d'arachide raffinée	Refined peanut oil
57	Huile de coton	Cottonseed oil

58	Huile de soja / végétale (e.g., Ninaa)	Soybean / vegetable oil (e.g., Ninaa)
59	Autres huiles n.d.a. (maïs, soja, huile palmiste, etc.)	Other oils n.e.s. (corn, soybean, palm kernel oil, etc.)
60	Mangue	Mango
61	Ananas	Pineapple
62	Orange	Orange
63	Banane douce	Sweet banana
64	Citrons	Lemons
65	Autres agrumes	Other citrus
66	Avocats	Avocados
67	Pastèque, Melon	Watermelon, Melon
68	Dattes	Dates
69	Noix de coco	Coconut
70	Canne à sucre	Sugar cane
71	Autres fruits (pommes, raisin, etc.)	Other fruits (apples, grapes, etc.)
72	Salade (laitue, batavia, etc.)	Salad (lettuce, batavia, etc.)
73	Choux	Cabbage
74	Carotte	Carrot
75	Haricot vert	Green bean
76	Concombre	Cucumber
77	Aubergine, Courge/Courgette	Eggplant, Squash / Zucchini
78	Poivron frais	Fresh bell pepper
79	Tomate fraîche	Fresh tomato
80	Tomate séchée	Dried tomato
81	Gombo frais	Fresh okra
82	Gombo sec	Dry okra
83	Oignon frais	Fresh onion
84	Ail	Garlic
85	Feuilles d'oseille (bissap)	Sorrel leaves (bissap)
86	Feuilles de baobab/ "laalo"	Baobab leaves / "laalo"
87	Feuilles de haricot/ "niébé"	Bean leaves / "cowpea"
88	Feuilles nébédaye (moringa)	Nebedaye leaves (moringa)

89	Autres légumes en feuilles (manioc, taro, etc.)	Other leafy vegetables (cassava, taro, etc.)
90	Autre légumes frais n.d.a. (y compris fleurs blanches de bissap)	Other fresh vegetables n.e.s. (including white bissap flowers)
91	Concentré de tomate	Tomato concentrate
92	Petits pois conserve	Canned peas
93	Petit pois secs	Dry peas
94	Autres légumes secs n.d.a.	Other pulses n.e.s.
95	Niébé/Haricots secs	Cowpea / Dried beans
96	Arachides fraîches en coques	Fresh peanuts in shell
97	Arachides séchées en coques	Dried peanuts in shell
98	Arachides décortiquées ou pilées (noflaye)	Peanuts, shelled or crushed (noflaye)
99	Arachide grillée	Roasted peanut
100	Pâte d'arachide	Peanut paste
101	Sésame	Sesame
102	Noix de cajou	Cashew nut
104	Manioc	Cassava
105	Igname	Yam
106	Plantain	Plantain
107	Pomme de terre	Potato
108	Taro (diabéré), macabo	Taro (diabetic), macabo
109	Patate douce	Sweet potato
110	Autres tubercules n.d.a.	Other tubers n.e.s.
111	Farines de manioc	Cassava flour
112	Gari, tapioca	Gari, tapioca
113	Attiéke	Attiéke
114	Sucre (poudre ou morceaux)	Sugar (powder or lumps)
115	Miel	Honey
116	Chocolat à croquer, pâte à tartiner	Chewable chocolate, spread
117	Caramel, bonbons, confiseries, etc.	Caramel, candy, confectionery, etc.
118	Sel	Salt
119	Piment	chilli pepper

120	Gingembre	Ginger
121	Cube alimentaire (Maggi, Jumbo,)	Food cube (Maggi, Jumbo,)
122	Arôme (Maggi, Jumbo, etc.)	Aroma (Maggi, Jumbo, etc.)
123	Soumbala (moutarde africaine)	Soumbala (African mustard)
124	Mayonnaise	Mayonnaise
125	Vinaigre /moutarde	Vinegar / mustard
126	Autres condiments (poivre etc.)	Other condiments (pepper etc.)
127	Noix de cola	Cola nuts
128	Autres produits alimentaires	Other food products
129	Café	Coffee
130	Thé	Tea
131	Chocolat en poudre	Chocolate powder
132	Autres tisanes et infusions n.d.a. (quinquelibat, citronelle, etc.)	Other herbal teas and infusions n.e.s. (quinquelibat, citronella, etc.)
133	Jus de fruits (orange, bissap, gingembre, jus de cajou,etc.)	Fruit juice (orange, bissap, ginger, cashew juice, etc.)
134	Eau minérale filtrée	Filtered mineral water
135	Boissons gazeuses (coca, etc.)	Soft drinks (cola, etc.)
136	Jus en poudre	Powdered juice
137	Bières et vins traditionnels (soumsoum, sung, vin de palme, vin de cajou, etc. ..)	Traditional beers and wines (soumsoum, sung, palm wine, cashew wine, etc.)
138	Bières industrielles	Industrial beers
139	Huile de palme raffinée	Refined palm oil
140	Huile d'arachide	Peanut oil

Supplemental Table S2. Energy and micronutrient requirements

	Energy (kcal/d)[1]	Vitamin A (µg RAE/d)	Thiamin (mg/d)	Riboflavin (mg/d)	Niacin (mg/d)	Folate (µg DFE/d)	Vitamin B12 (µg/d)	Iron[2] (mg/d)	Absorbed zinc (mg/d)
Children 6 mo – 3 years	969	210	0.4	0.4	5	120	0.7	n/a	1.074
Children 4 years	1,300	275	0.5	0.5	6	160	1	n/a	1.39
NPNL WRA, 15-17 years	2,500	485	0.9	0.9	11	330	2	n/a	2.89
NPNL WRA, 18-49 years	2,131	500	0.9	0.9	11	320	2	n/a	2.89
Pregnant WRA, 15-17 years	2,916	530	1.2	1.2	14	520	2.2	40	3.59
Pregnant WRA, 19-49 years	2,548	550	1.2	1.2	14	520	2.2	40	3.59

NPLN, non-pregnant non-lactating; WRA, women of reproductive age.

[1]Energy requirements were estimated based on FAO human energy requirements assuming moderate physical activity levels for children age 12-59 months and physical activity levels corresponding to 1.6 x basal metabolic rate for adults (Food and Agricultural Organization of the United Nations & World Health Organization, 2004). Energy requirements for each target group averaged over the age- and sex-specific requirements for that group. Energy requirements for pregnant women based on assumed average energy requirement during pregnancy of 417.5 kcal/day (average of additional 360 kcal/day and 475 kcal/day during the second and third trimesters, respectively) (Food and Agricultural Organization of the United Nations & World Health Organization, 2004).

[2]The iron requirement for pregnant women based on assuming 10% bioavailability of iron. For children and non-pregnant women of reproductive age, we used the full probably method (assuming 10% bioavailability) to assess the adequacy of the household diet for meeting iron needs (World Health Organization & Food and Agricultural Organization, 2006).

Supplemental Table S3. Existing and hypothetical large-scale food fortification standards

Food vehicle	Vitamin A	Thiamin	Riboflavin	Niacin	Folic acid	Vitamin B12	Iron	Zinc
	**Mandated fortification level (mg/kg)**							
Refined oil	17.5				N/A	N/A	N/A	N/A
Wheat flour	N/A				2.5	N/A	60	N/A
	**Hypothetical fortification level (mg/kg)**							
Wheat flour[1]	N/A	3	2	40	5	0.02	N/A	55
Rice[2]	3.1	9.7		109.2	2.5	0.02	48	72

N/A, not applicable (nutrient not in standard for the given food); WFP, World Food Programme.

[1]Based on 2022 WHO wheat flour fortification guidelines assuming 75-149 g/day wheat flour consumption (World Health Organization, 2022).

[2]Based on the World Food Programme (WFP) technical specifications for fortified rice.

Supplemental Table S4. Modeling assumptions and scenarios

	Refined oil	Wheat flour	Rice
**Current compliance**	• 95% of refined oil is fortifiable[1]<br><br>• Of fortifiable refined oil, 59% is fortified (to any level)[3]<br><br>• Average vitamin content in fortified refined oil as a percent of the standard, adjusted for expected losses: 82%[3,4]	• 100% of wheat flour is fortifiable[2]<br><br>• Of fortifiable wheat flour, 76% fortified (to any level)[3]<br><br>• Average micronutrient content in fortified wheat flour as a percent of the standard, adjusted for expected losses: 43% [3,4]	Not Applicable
**Realistic improved compliance (oil); Realistic compliance (rice)**	• 95% of refined oil is fortifiable[1]<br><br>• Of fortifiable refined oil, 75% is fortified (to any level)[5]<br><br>• Average vitamin A content in fortified refined oil as a percent of the standard, adjusted for expected losses: 100%[6]	• 100% of wheat flour is fortifiable[2]<br><br>• Of fortifiable wheat flour, 76% fortified (to any level)[3]<br><br>• Average micronutrient content in fortified wheat flour as a percent of the standard, adjusted for expected losses: 100%[6]	• 60% of rice is fortifiable (imported rice only)[1,7]<br><br>• Of fortifiable rice, 75% is fortified (to any level)[5]<br><br>• Average micronutrient content among fortified rice as a percent of the hypothetical standard, adjusted for expected losses: 100%[6]

Fortifiable: a food vehicle that is industrially processed

[1] Assessing Large-Scale Food Fortification Opportunities in Senegal (USAID Advancing Food Fortification Opportunities to Reinforce Diets (USAID AFFORD), 2023a)

[2] USAID AFFORD Market Assessment for Fortifiable Foods in Senegal (USAID Advancing Food Fortification Opportunities to Reinforce Diets (USAID AFFORD), 2023b).

[3]Based on qualitative and quantitative testing of market samples as reported in USAID AFFORD Market Assessment for Fortifiable Foods in Senegal (USAID Advancing Food Fortification Opportunities to Reinforce Diets (USAID AFFORD), 2023b). Note that modeled contents of all micronutrients in fortified wheat flour based on average iron contents from quantitative testing (26 mg/kg, or ~43% of the national standard of 60 mg/kg).

[4]Modeling assumes that average micronutrient contents at homes is similar to average contents at markets.

[5]Modeling assumption.

[6]Modeling assumption calculated as mandatory or hypothetical fortification level (Supplemental Table S2) less expected losses (Supplemental Table S4).

[7] Currently, ~60% of the rice supply in Senegal comes from imported rice. We assumed all imported rice was potentially fortifiable. A negligible share of the rice supply (~1.5%) is domestically refined at industrial scale (and hence may be feasible for fortification). We did not account for this 1.5% in the rice fortification modeling.

Supplemental Table S5. Assumed micronutrient retention in fortified foods

	Assumed micronutrient retention in fortified foods (%)							
**Food vehicle**	**Vitamin A**	**Thiamin**	**Riboflavin**	**Niacin**	**Folic acid**	**Vitamin B12**	**Iron**	**Zinc**
Refined oil[1]	70%							
Wheat flour[1]		80%	80%	80%	80%	80%	100%	100%
Rice[2]	50%	80%	80%	80%	80%	80%	83%	82%

[1]Oil and wheat flour retention rates based on expert opinion.

[2]Rice retention rates based on average micronutrient retention in fortified rice kernels produced using hot extrusion (estimated from Figure 2 in Pyo, Tsang & Parker (2022)) and expert opinion. Retention rates could be higher if fortified rice kernel producers included overages to offset losses during the hot extrusion process.

Supplemental Table S6. Tolerable upper intake levels

	Preformed retinol (µg /d)	Thiamin (mg/d)	Riboflavin (mg/d)	Niacin (mg/d)	Folic acid (µg /d)	Vitamin B12 (µg/d)	Iron (mg/d)	Zinc (mg/d)
Children 6 mo – 3 years	600	n/a	n/a	n/a	300	n/a	40	7
Children 4 years	900	n/a	n/a	n/a	400	n/a	40	10
NPNL WRA, 15-17 years	2800	n/a	n/a	n/a	800	n/a	45	40
NPNL WRA, 18-49 years	3000	n/a	n/a	n/a	1000	n/a	45	40
Pregnant WRA, 15-17 years	2800	n/a	n/a	n/a	800	n/a	45	40
Pregnant WRA, 19-49 years	3000	n/a	n/a	n/a	1000	n/a	45	40

NPLN, non-pregnant non-lactating; WRA, women of reproductive age.

Supplemental Table S7. Contribution of LSFF to meeting the vitamin A requirements of women of reproductive age and children

		Prevalence of apparent inadequacy (%)					
		Baseline diets	Refined oil current compliance	Refined oil improved compliance	Rice	Refined oil current compliance + rice	Refined oil improved compliance + rice
		Women of reproductive age					
National	Senegal	70	35	24	55	24	16
Residence	Urban	59	18	10	42	11	6
	Rural	82	54	40	69	38	27
Region	Dakar	54	14	8	36	8	5
	Ziguinchor	52	35	26	30	19	15
	Diourbel	84	52	41	75	41	33
	Saint-Louis	91	33	11	89	30	10
	Tambacounda	78	64	52	65	48	39
	Kaolack	67	40	30	47	23	17
	Thies	77	28	15	63	22	12
	Louga	91	47	23	79	31	16
	Fatick	74	48	34	53	26	16
	Kolda	54	44	40	36	27	24
	Matam	88	48	25	78	30	13
	Kaffrine	79	59	48	62	44	36
	Kedougou	62	50	44	46	31	28
	Sedhiou	62	48	41	34	20	16
		Children 6-59 months					
National	Senegal	70	37	26	54	24	17
Residence	Urban	58	18	10	40	9	5
	Rural	80	53	39	66	36	27
Region	Dakar	53	13	7	34	5	3
	Ziguinchor	50	35	28	31	22	17
	Diourbel	80	52	41	69	41	33
	Saint-Louis	90	31	15	87	28	14
	Tambacounda	78	65	52	64	46	37
	Kaolack	66	42	31	44	23	18
	Thies	76	31	20	64	23	14
	Louga	88	42	21	75	27	15
	Fatick	76	49	38	51	28	23
	Kolda	52	42	37	34	24	21
	Matam	87	46	25	76	27	13
	Kaffrine	82	58	47	61	37	31
	Kedougou	57	45	41	41	32	30
	Sedhiou	58	46	37	32	20	16

WRA, women of reproductive age.

Supplemental Table S8. Risk of high vitamin A intakes among women of reproductive age and children

		Risk of high intakes (%)					
		Baseline diets	Refined oil current compliance	Refined oil improved compliance	Rice	Refined oil current compliance + rice	Refined oil improved compliance + rice
		Women of reproductive age					
National	Senegal	0	0	0	0	0	0
Residence	Urban	1	1	1	1	1	1
	Rural	0	0	0	0	0	0
Region	Dakar	1	1	1	1	1	1
	Ziguinchor	0	0	0	0	0	0
	Diourbel	0	0	0	0	0	0
	Saint-Louis	0	0	0	0	0	0
	Tambacounda	0	0	0	0	0	0
	Kaolack	0	0	0	0	0	0
	Thies	0	0	0	0	0	0
	Louga	0	0	0	0	0	0
	Fatick	0	0	0	0	0	0
	Kolda	0	0	0	0	0	0
	Matam	0	0	0	0	0	0
	Kaffrine	0	0	0	0	0	0
	Kedougou	0	0	0	0	0	0
	Sedhiou	0	0	0	0	0	0
		Children 6-59 months					
National	Senegal	1	2	2	1	2	2
Residence	Urban	2	3	4	3	3	5
	Rural	0	0	0	0	0	0
Region	Dakar	4	6	8	5	6	9
	Ziguinchor	0	1	1	0	1	1
	Diourbel	0	0	0	0	0	0
	Saint-Louis	0	0	1	0	0	1
	Tambacounda	0	0	0	0	0	0
	Kaolack	0	0	0	0	0	1
	Thies	0	1	1	0	1	1
	Louga	0	0	0	0	0	0
	Fatick	0	0	0	0	0	0
	Kolda	1	1	1	1	1	1
	Matam	0	0	1	0	1	1
	Kaffrine	0	0	0	0	0	1
	Kedougou	0	1	2	0	1	3
	Sedhiou	0	0	0	0	1	1

WRA, women of reproductive age.

Supplemental Table S9. Contribution of LSFF plus high-dose vitamin A supplementation to meeting the vitamin A requirements children

		**Prevalence of apparent inadequacy (%)**						
		Baseline diets	VAS[1]	Refined oil current compliance + VAS	Refined oil improved compliance + VAS	Rice + VAS	Refined oil current compliance + rice + VAS	Refined oil improved compliance + rice + VAS
		Children 6-59 months						
National	Senegal	70	48	27	20	37	18	13
Residence	Urban	58	25	8	5	18	4	3
	Rural	80	66	42	31	53	28	21
Region	Dakar	53	27	8	4	18	3	2
	Ziguinchor	50	32	23	18	19	12	9
	Diourbel	80	59	37	30	49	29	24
	Saint-Louis	90	74	25	12	71	23	11
	Tambacounda	78	61	49	42	49	36	29
	Kaolack	66	45	31	24	28	16	12
	Thies	76	39	20	15	34	16	11
	Louga	88	74	35	18	63	22	13
	Fatick	76	50	35	29	35	21	16
	Kolda	52	37	31	28	25	18	16
	Matam	87	73	39	21	61	22	10
	Kaffrine	82	56	42	34	42	25	21
	Kedougou	57	45	36	34	34	27	26
	Sedhiou	58	44	33	27	25	16	13

VAS, vitamin A supplementation; WRA, women of reproductive age.

[1]VAS coverage based on 2019 DHS data.

Supplemental Table S10. Predicted overlap of inadequate, adequate, and high vitamin A intakes with high-dose vitamin A supplementation among children 6-59 months of age

			LSFF modeling scenario				
		**Overlap between dietary vitamin A adequacy and VAS receipt**	**Refined oil current compliance**	**Refined oil improved compliance**	**Rice**	**Refined oil current compliance + rice**	**Refined oil improved compliance + rice**
**National**	Senegal	VA intake above the UL and VAS[1] (%)	1	1	1	1	1
		Adequate dietary VA and VAS[2] (%)	30	33	23	34	35
		Inadequate dietary VA and VAS[3] (%)	11	8	18	7	5
		VA intake above the UL and no VAS[4] (%)	1	1	1	1	1
		Adequate dietary VA and no VAS[5] (%)	31	38	22	40	45
		Inadequate dietary VA and no VAS[6] (%)	26	19	36	17	12
**Residence**	Urban	VA intake above the UL and VAS[1] (%)	2	2	1	2	3
		Adequate dietary VA and VAS[2] (%)	51	55	39	56	57
		Inadequate dietary VA and VAS[3] (%)	10	6	23	5	3
		VA intake above the UL and no VAS[4] (%)	1	2	1	2	2
		Adequate dietary VA and no VAS[5] (%)	28	30	19	31	33
		Inadequate dietary VA and no VAS[6] (%)	8	5	17	4	2
	Rural	VA intake above the UL and VAS[1] (%)	0	0	0	0	0
		Adequate dietary VA and VAS[2] (%)	13	16	10	16	18
		Inadequate dietary VA and VAS[3] (%)	12	9	15	8	7
		VA intake above the UL and no VAS[4] (%)	0	0	0	0	0
		Adequate dietary VA and no VAS[5] (%)	34	45	24	47	54
		Inadequate dietary VA and no VAS[6] (%)	41	30	51	28	21
**Region**	Dakar	VA intake above the UL and VAS[1] (%)	3	4	2	3	4
		Adequate dietary VA and VAS[2] (%)	45	47	35	49	48
		Inadequate dietary VA and VAS[3] (%)	6	3	16	2	1
		VA intake above the UL and no VAS[4] (%)	3	4	3	3	4
		Adequate dietary VA and no VAS[5] (%)	36	39	26	40	41
		Inadequate dietary VA and no VAS[6] (%)	8	4	18	3	2
	Ziguinchor	VA intake above the UL and VAS[1] (%)	1	1	0	1	1
		Adequate dietary VA and VAS[2] (%)	39	42	39	44	45
		Inadequate dietary VA and VAS[3] (%)	15	13	15	10	9
		VA intake above the UL and no VAS[4] (%)	0	0	0	0	0
		Adequate dietary VA and no VAS[5] (%)	25	29	29	34	37

		Inadequate dietary VA and no VAS[6] (%)	20	16	16	11	8
	Diourbel	VA intake above the UL and VAS[1] (%)	0	0	0	0	0
		Adequate dietary VA and VAS[2] (%)	14	17	8	17	19
		Inadequate dietary VA and VAS[3] (%)	15	12	21	12	10
		VA intake above the UL and no VAS[4] (%)	0	0	0	0	0
		Adequate dietary VA and no VAS[5] (%)	34	41	22	42	47
		Inadequate dietary VA and no VAS[6] (%)	36	29	48	29	24
	Saint-Louis	VA intake above the UL and VAS[1] (%)	0	0	0	0	1
		Adequate dietary VA and VAS[2] (%)	15	18	4	16	18
		Inadequate dietary VA and VAS[3] (%)	6	3	18	6	3
		VA intake above the UL and no VAS[4] (%)	0	0	0	0	0
		Adequate dietary VA and no VAS[5] (%)	53	66	9	55	67
		Inadequate dietary VA and no VAS[6] (%)	25	12	69	23	11
	Tambacounda	VA intake above the UL and VAS[1] (%)	0	0	0	0	0
		Adequate dietary VA and VAS[2] (%)	22	28	20	30	31
		Inadequate dietary VA and VAS[3] (%)	18	13	20	11	9
		VA intake above the UL and no VAS[4] (%)	0	0	0	0	0
		Adequate dietary VA and no VAS[5] (%)	13	20	15	25	32
		Inadequate dietary VA and no VAS[6] (%)	46	39	44	35	28
	Kaolack	VA intake above the UL and VAS[1] (%)	0	0	0	0	1
		Adequate dietary VA and VAS[2] (%)	31	36	28	38	38
		Inadequate dietary VA and VAS[3] (%)	13	9	17	7	6
		VA intake above the UL and no VAS[4] (%)	0	0	0	0	0
		Adequate dietary VA and no VAS[5] (%)	26	32	28	39	43
		Inadequate dietary VA and no VAS[6] (%)	29	23	27	16	12
	Thies	VA intake above the UL and VAS[1] (%)	0	1	0	1	1
		Adequate dietary VA and VAS[2] (%)	45	51	26	49	53
		Inadequate dietary VA and VAS[3] (%)	13	6	32	8	4
		VA intake above the UL and no VAS[4] (%)	0	0	0	0	0
		Adequate dietary VA and no VAS[5] (%)	23	28	10	27	31
		Inadequate dietary VA and no VAS[6] (%)	19	14	32	15	10
	Louga	VA intake above the UL and VAS[1] (%)	0	0	0	0	0
		Adequate dietary VA and VAS[2] (%)	11	15	5	13	16
		Inadequate dietary VA and VAS[3] (%)	8	3	14	5	2
		VA intake above the UL and no VAS[4] (%)	0	0	0	0	0
		Adequate dietary VA and no VAS[5] (%)	47	64	20	60	69

		Inadequate dietary VA and no VAS[6] (%)	35	18	62	22	13
	Fatick	VA intake above the UL and VAS[1] (%)	0	0	0	0	0
		Adequate dietary VA and VAS[2] (%)	28	34	25	36	36
		Inadequate dietary VA and VAS[3] (%)	14	9	18	7	7
		VA intake above the UL and no VAS[4] (%)	0	0	0	0	0
		Adequate dietary VA and no VAS[5] (%)	22	28	23	36	41
		Inadequate dietary VA and no VAS[6] (%)	35	29	34	21	16
	Kolda	VA intake above the UL and VAS[1] (%)	0	0	0	0	0
		Adequate dietary VA and VAS[2] (%)	31	33	34	37	38
		Inadequate dietary VA and VAS[3] (%)	13	11	10	6	5
		VA intake above the UL and no VAS[4] (%)	0	0	0	0	0
		Adequate dietary VA and no VAS[5] (%)	27	30	32	38	40
		Inadequate dietary VA and no VAS[6] (%)	29	26	24	18	16
	Matam	VA intake above the UL and VAS[1] (%)	0	0	0	0	0
		Adequate dietary VA and VAS[2] (%)	16	19	7	18	20
		Inadequate dietary VA and VAS[3] (%)	7	4	16	5	3
		VA intake above the UL and no VAS[4] (%)	0	0	0	0	1
		Adequate dietary VA and no VAS[5] (%)	38	56	17	54	65
		Inadequate dietary VA and no VAS[6] (%)	39	20	59	22	10
	Kaffrine	VA intake above the UL and VAS[1] (%)	0	0	0	0	0
		Adequate dietary VA and VAS[2] (%)	24	26	21	29	31
		Inadequate dietary VA and VAS[3] (%)	18	16	21	13	11
		VA intake above the UL and no VAS[4] (%)	0	0	0	0	1
		Adequate dietary VA and no VAS[5] (%)	18	26	18	33	37
		Inadequate dietary VA and no VAS[6] (%)	39	31	40	24	20
	Kedougou	VA intake above the UL and VAS[1] (%)	1	1	0	1	1
		Adequate dietary VA and VAS[2] (%)	17	19	19	22	22
		Inadequate dietary VA and VAS[3] (%)	10	8	9	5	5
		VA intake above the UL and no VAS[4] (%)	0	1	0	0	2
		Adequate dietary VA and no VAS[5] (%)	37	38	40	46	45
		Inadequate dietary VA and no VAS[6] (%)	35	33	33	27	25
	Sedhiou	VA intake above the UL and VAS[1] (%)	0	0	0	0	0
		Adequate dietary VA and VAS[2] (%)	23	27	29	32	33
		Inadequate dietary VA and VAS[3] (%)	13	9	7	4	3
		VA intake above the UL and no VAS[4] (%)	0	0	0	0	0
		Adequate dietary VA and no VAS[5] (%)	30	36	39	47	50

		Inadequate dietary VA and no VAS[6] (%)	33	27	24	16	13

UL, tolerable upper intake level; VA, vitamin A; VAS, high-dose vitamin A supplementation.

[1]Children at risk of high vitamin A intake with LSFF and also receive VAS.

[2]Children with adequate (but not high) vitamin A intake with LSFF and also receive VAS.

[3]Children with inadequate vitamin A intake with LSFF and receive VAS.

[4]Cildren at risk of high vitamin A intake with LSFF and do not receive VAS.

[5]Children with adequate (but not high) vitamin A intake with LSFF and do not receive VAS.

[6]Children with inadequate vitamin A intake with LSFF and do not VAS.

Note: VAS coverage based on 2019 DHS data.

Supplemental Table S11. Contribution of LSFF to meeting the thiamin requirements of women of reproductive age and children

		Prevalence of apparent inadequacy (%)					
		Baseline diets	Wheat flour current compliance	Wheat flour improved compliance	Rice	Wheat flour current compliance + rice	Wheat flour improved compliance + rice
		Women of reproductive age					
National	Senegal	92	89	84	47	45	43
Residence	Urban	95	93	87	45	43	41
	Rural	88	86	82	48	47	45
Region	Dakar	96	93	86	41	40	37
	Ziguinchor	98	97	94	41	41	39
	Diourbel	94	90	86	65	63	60
	Saint-Louis	99	97	95	89	87	85
	Tambacounda	82	79	76	36	35	33
	Kaolack	88	86	80	23	22	20
	Thies	94	92	88	64	62	59
	Louga	93	89	84	53	51	48
	Fatick	84	82	77	21	20	19
	Kolda	85	83	80	29	28	27
	Matam	89	83	74	50	46	41
	Kaffrine	79	75	69	28	27	25
	Kedougou	77	75	70	33	33	31
	Sedhiou	96	95	92	27	26	26
		Children 6-59 months					
National	Senegal	86	82	75	44	42	38
Residence	Urban	92	87	79	43	41	37
	Rural	82	79	72	44	42	39
Region	Dakar	94	89	81	40	38	35
	Ziguinchor	96	93	90	40	39	37
	Diourbel	89	85	75	61	58	51
	Saint-Louis	97	95	89	87	85	80
	Tambacounda	74	72	68	33	32	31
	Kaolack	78	75	67	19	18	16
	Thies	88	83	77	59	55	51
	Louga	88	85	78	51	49	45
	Fatick	78	74	67	19	17	16
	Kolda	77	74	71	23	23	21
	Matam	80	73	64	45	41	34
	Kaffrine	67	61	55	24	24	20
	Kedougou	69	66	63	32	31	29
	Sedhiou	93	92	86	28	28	27

WRA, women of reproductive age.

Supplemental Table S12. Contribution of LSFF to meeting the riboflavin requirements of women of reproductive age and children

		**Prevalence of apparent inadequacy (%)**		
		Baseline diets	Wheat flour current compliance	Wheat flour improved compliance
		Women of reproductive age		
National	Senegal	95	94	92
Residence	Urban	91	89	86
	Rural	99	98	98
Region	Dakar	86	83	79
	Ziguinchor	97	96	95
	Diourbel	100	99	98
	Saint-Louis	98	98	96
	Tambacounda	100	100	99
	Kaolack	98	97	96
	Thies	97	96	95
	Louga	98	98	97
	Fatick	99	97	97
	Kolda	96	95	95
	Matam	97	97	94
	Kaffrine	99	99	98
	Kedougou	98	97	97
	Sedhiou	98	96	95
		Children 6-59 months		
National	Senegal	94	92	90
Residence	Urban	89	87	83
	Rural	98	97	95
Region	Dakar	84	80	76
	Ziguinchor	95	94	93
	Diourbel	98	97	95
	Saint-Louis	98	96	94
	Tambacounda	99	99	98
	Kaolack	96	96	94
	Thies	95	94	92
	Louga	98	97	95
	Fatick	99	99	95
	Kolda	95	94	94
	Matam	93	92	87
	Kaffrine	98	97	97
	Kedougou	96	95	95
	Sedhiou	96	96	95

WRA, women of reproductive age.

Supplemental Table S13. Contribution of LSFF to meeting the folate requirements of women of reproductive age and children

		Prevalence of apparent inadequacy (%)									
		Baseline diets	Wheat flour current compliance	Wheat flour improved complianc e	Wheat flour current compliance (5 mg/kg)[1]	Wheat flour improved compliance (5 mg/kg)	Rice	Wheat flour current compliance + rice	Wheat flour improved complianc e + rice	Wheat flour current compliance (5 mg/kg) + rice	Wheat flour improved compliance (5 mg/kg) + rice
		Women of reproductive age									
National	Senegal	92	82	53	61	25	47	42	28	31	13
Residence	Urban	97	87	53	63	20	47	41	26	30	10
	Rural	86	76	53	59	30	47	42	29	33	15
Region	Dakar	98	88	51	62	18	43	37	24	27	9
	Ziguinchor	98	93	71	78	37	40	38	30	32	17
	Diourbel	89	74	41	50	15	63	53	29	35	9
	Saint-Louis	99	94	69	77	30	89	85	63	70	28
	Tambacounda	85	78	63	68	43	38	34	28	30	20
	Kaolack	83	70	48	52	29	21	17	12	14	6
	Thies	94	86	53	62	18	63	58	37	43	12
	Louga	93	84	55	62	25	54	48	31	36	15
	Fatick	74	60	38	44	23	17	13	9	10	5
	Kolda	88	82	65	69	47	32	29	24	25	19
	Matam	97	84	53	58	29	56	47	27	29	13
	Kaffrine	70	59	39	43	23	27	22	15	17	9
	Kedougou	91	88	71	77	51	42	41	35	38	27
	Sedhiou	90	81	65	69	55	25	23	21	21	19
		Children 6-59 months									
National	Senegal	76	56	31	35	18	39	29	16	18	9
Residence	Urban	87	63	31	36	16	40	29	14	17	8
	Rural	68	51	32	35	20	39	29	18	19	11
Region	Dakar	91	67	31	36	14	37	27	13	15	7
	Ziguinchor	88	74	49	53	30	38	34	23	26	17
	Diourbel	73	49	25	29	11	53	35	18	20	7
	Saint-Louis	95	75	41	47	21	84	69	37	43	18
	Tambacounda	68	57	39	41	30	26	22	16	16	11
	Kaolack	55	39	24	26	17	12	9	4	5	3
	Thies	80	59	31	35	17	53	40	23	24	12

	Louga	83	56	30	35	17	47	30	17	19	10
	Fatick	44	28	16	18	11	9	7	3	4	3
	Kolda	70	59	43	47	32	23	20	15	16	9
	Matam	86	60	35	39	22	49	32	17	19	9
	Kaffrine	40	28	17	18	13	17	11	8	8	5
	Kedougou	76	64	50	53	38	36	31	25	27	20
	Sedhiou	73	55	44	46	37	22	19	17	17	16

WRA, women of reproductive age.

[1]Based on wheat flour consumption patterns in Senegal, the current global guidance from WHO (2022) suggests wheat flour be fortified with 5 mg/kg folic acid (compared to 2.5 mg/kg in Senegal's national wheat flour fortification standard).

Supplemental Table S14. Risk of high folic acid intakes among women of reproductive age and children

		Prevalence of apparent inadequacy (%)									
		Baseline diets	Wheat flour current compliance	Wheat flour improved compliance	Wheat flour current compliance (5 mg/kg)[1]	Wheat flour improved compliance (5 mg/kg)	Rice	Wheat flour current compliance + rice	Wheat flour improved compliance + rice	Wheat flour current compliance (5 mg/kg) + rice	Wheat flour improved compliance (5 mg/kg) + rice
		Women of reproductive age									
National	Senegal	0	0	0	0	0	0	0	0	0	1
Residence	Urban	0	0	0	0	0	0	0	0	0	1
	Rural	0	0	0	0	0	0	0	0	0	1
Region	Dakar	0	0	0	0	0	0	0	0	0	1
	Ziguinchor	0	0	0	0	0	1	1	1	1	3
	Diourbel	0	0	0	0	0	0	0	0	0	0
	Saint-Louis	0	0	0	0	0	0	0	0	0	0
	Tambacounda	0	0	0	0	0	0	0	0	0	0
	Kaolack	0	0	0	0	0	0	0	0	0	0
	Thies	0	0	0	0	0	0	0	0	0	1
	Louga	0	0	0	0	0	0	0	0	0	0
	Fatick	0	0	0	0	0	0	0	0	0	0
	Kolda	0	0	0	0	0	2	2	3	2	3
	Matam	0	0	0	0	0	0	0	0	0	1
	Kaffrine	0	0	0	0	0	0	0	0	0	1
	Kedougou	0	0	0	0	0	0	0	0	0	1
	Sedhiou	0	0	0	0	0	2	2	3	3	3
		Children 6-59 months									
National	Senegal	0	0	0	0	0	1	2	3	3	7
Residence	Urban	0	0	0	0	0	1	2	3	3	8
	Rural	0	0	0	0	0	2	2	3	3	6
Region	Dakar	0	0	0	0	0	1	1	2	2	8
	Ziguinchor	0	0	0	0	0	8	10	15	14	24
	Diourbel	0	0	0	0	0	0	0	1	1	3
	Saint-Louis	0	0	0	0	0	0	0	0	0	1
	Tambacounda	0	0	0	0	0	3	3	4	4	6
	Kaolack	0	0	0	0	1	0	0	2	1	6
	Thies	0	0	0	0	1	0	0	0	0	2

	Louga	0	0	0	0	0	0	0	0	0	2
	Fatick	0	0	0	0	1	0	0	1	1	4
	Kolda	0	0	0	0	0	8	9	10	10	14
	Matam	0	0	0	0	1	0	0	1	1	7
	Kaffrine	0	0	0	0	0	1	1	2	1	4
	Kedougou	0	0	0	0	0	2	3	4	4	7
	Sedhiou	0	0	0	0	0	10	11	15	13	21

WRA, women of reproductive age.

[1]Based on wheat flour consumption patterns in Senegal, the current global guidance from WHO (2022) suggests wheat flour be fortified with 5 mg/kg folic acid (compared to 2.5 mg/kg in Senegal's national wheat flour fortification standard).

Supplemental Table S15. Contribution of LSFF to meeting the vitamin B12 requirements of women of reproductive age and children

		Prevalence of apparent inadequacy (%)					
		Baseline diets	Wheat flour current compliance	Wheat flour improved compliance	Rice	Wheat flour current compliance + rice	Wheat flour improved compliance + rice
		Women of reproductive age					
National	Senegal	29	24	19	15	12	9
Residence	Urban	20	14	8	9	6	3
	Rural	40	36	32	21	18	15
Region	Dakar	16	9	4	7	4	1
	Ziguinchor	54	46	34	24	21	16
	Diourbel	14	12	8	8	6	4
	Saint-Louis	34	26	19	30	23	16
	Tambacounda	68	65	59	38	35	32
	Kaolack	27	23	19	7	6	5
	Thies	14	10	8	10	7	5
	Louga	26	20	16	16	12	9
	Fatick	35	32	29	10	9	8
	Kolda	66	62	58	26	25	23
	Matam	40	33	25	24	19	13
	Kaffrine	40	38	33	15	14	12
	Kedougou	80	77	72	46	43	42
	Sedhiou	69	62	55	21	19	18
		Children 6-59 months					
National	Senegal	25	21	17	12	9	8
Residence	Urban	14	9	6	6	3	2
	Rural	34	30	26	16	14	13
Region	Dakar	10	5	3	5	2	1
	Ziguinchor	39	28	24	19	14	13
	Diourbel	9	7	6	3	3	2
	Saint-Louis	26	20	14	23	17	12
	Tambacounda	61	56	50	32	30	27
	Kaolack	21	18	15	5	4	4
	Thies	13	10	8	9	6	5
	Louga	20	17	12	13	10	7
	Fatick	30	27	24	9	9	9
	Kolda	56	53	46	22	21	18
	Matam	33	26	21	17	12	10
	Kaffrine	40	36	30	12	10	9
	Kedougou	76	72	65	44	42	39
	Sedhiou	56	50	42	19	18	17

WRA, women of reproductive age.

Supplemental Table S16. Contribution of LSFF to meeting the iron requirements of women of reproductive age and children

		**Prevalence of apparent inadequacy (%)**					
		Baseline diets	Wheat flour current compliance	Wheat flour improved compliance	Rice	Wheat flour current compliance + rice	Wheat flour improved compliance + rice
		Women of reproductive age					
National	Senegal	61	54	46	44	39	33
Residence	Urban	63	55	46	44	38	32
	Rural	58	52	46	43	39	35
Region	Dakar	67	57	46	47	40	33
	Ziguinchor	67	60	51	41	37	32
	Diourbel	61	52	42	48	41	34
	Saint-Louis	80	71	61	76	68	57
	Tambacounda	56	52	47	38	36	33
	Kaolack	54	49	43	33	30	27
	Thies	64	56	46	52	46	39
	Louga	69	61	51	52	46	39
	Fatick	51	45	39	31	28	25
	Kolda	53	50	45	34	32	29
	Matam	62	54	45	48	42	34
	Kaffrine	51	46	41	36	33	30
	Kedougou	55	51	46	38	36	33
	Sedhiou	58	53	48	32	30	28
		Children 6-59 months					
National	Senegal	43	38	33	30	27	23
Residence	Urban	46	41	34	32	28	23
	Rural	40	36	32	28	25	22
Region	Dakar	50	43	36	35	30	25
	Ziguinchor	47	42	36	28	25	21
	Diourbel	44	38	31	34	29	24
	Saint-Louis	59	52	44	56	49	42
	Tambacounda	38	35	31	25	23	20
	Kaolack	38	34	30	22	20	17
	Thies	46	40	34	37	33	28
	Louga	51	45	38	38	34	28
	Fatick	35	31	27	20	18	15
	Kolda	35	32	29	19	17	15
	Matam	45	39	33	34	29	24
	Kaffrine	33	30	26	22	19	17
	Kedougou	39	37	34	26	24	22
	Sedhiou	39	35	32	20	18	16

WRA, women of reproductive age.

Supplemental Table S17. Risk of high iron intakes among women of reproductive age and children

		Risk of high intakes (%)					
		Baseline diets	Wheat flour current compliance	Wheat flour improved compliance	Rice	Wheat flour current compliance + rice	Wheat flour improved compliance + rice
		Women of reproductive age					
National	Senegal	0	0	0	0	0	0
Residence	Urban	0	0	0	0	0	0
	Rural	0	0	0	0	0	0
Region	Dakar	0	0	0	0	0	0
	Ziguinchor	0	0	0	0	0	0
	Diourbel	0	0	0	0	0	0
	Saint-Louis	0	0	0	0	0	0
	Tambacounda	0	0	0	0	0	0
	Kaolack	0	0	0	0	0	0
	Thies	0	0	0	0	0	0
	Louga	0	0	0	0	0	0
	Fatick	0	0	0	0	0	0
	Kolda	0	0	0	0	0	0
	Matam	0	0	0	0	0	0
	Kaffrine	0	0	0	0	0	0
	Kedougou	2	2	2	2	2	2
	Sedhiou	0	0	0	0	0	0
		Children 6-59 months					
National	Senegal	0	0	0	0	0	0
Residence	Urban	0	0	0	0	0	0
	Rural	0	0	0	0	0	0
Region	Dakar	0	0	0	0	0	0
	Ziguinchor	0	0	0	0	0	0
	Diourbel	0	0	0	0	0	0
	Saint-Louis	0	0	0	0	0	0
	Tambacounda	0	0	0	0	0	0
	Kaolack	0	0	0	0	0	0
	Thies	0	0	0	0	0	0
	Louga	0	0	0	0	0	0
	Fatick	0	0	0	0	0	0
	Kolda	0	0	0	0	0	0
	Matam	0	0	0	0	0	0
	Kaffrine	0	0	0	0	0	0
	Kedougou	0	0	0	0	0	0
	Sedhiou	0	0	0	0	0	0

WRA, women of reproductive age.

Supplemental Table S18. Contribution of LSFF to meeting the zinc requirements of women of reproductive age and children

		**Prevalence of apparent inadequacy (%)**					
		Baseline diets	Wheat flour current compliance	Wheat flour improved compliance	Rice	Wheat flour current compliance + rice	Wheat flour improved compliance + rice
		Women of reproductive age					
National	Senegal	66	56	47	44	38	32
Residence	Urban	59	48	37	37	31	25
	Rural	74	65	57	51	45	40
Region	Dakar	52	40	31	31	25	21
	Ziguinchor	44	33	26	21	17	14
	Diourbel	73	59	47	55	44	35
	Saint-Louis	82	70	56	76	64	51
	Tambacounda	71	65	59	44	41	38
	Kaolack	72	64	57	39	36	33
	Thies	74	63	48	55	48	36
	Louga	80	70	59	55	48	44
	Fatick	69	61	55	35	31	29
	Kolda	61	56	52	35	33	31
	Matam	80	70	61	61	52	45
	Kaffrine	79	73	67	51	48	45
	Kedougou	62	56	50	34	31	29
	Sedhiou	68	62	57	30	29	28
		Children 6-59 months					
National	Senegal	74	67	61	57	52	48
Residence	Urban	75	65	58	56	50	46
	Rural	74	68	62	57	54	49
Region	Dakar	73	62	56	52	46	43
	Ziguinchor	59	50	44	35	33	30
	Diourbel	78	70	63	65	59	51
	Saint-Louis	89	83	72	84	79	68
	Tambacounda	70	66	62	53	50	48
	Kaolack	71	64	59	49	46	44
	Thies	80	71	64	65	58	54
	Louga	83	75	66	65	60	54
	Fatick	64	61	55	41	38	37
	Kolda	63	58	56	37	36	35
	Matam	87	79	72	70	64	58
	Kaffrine	72	69	65	57	54	51
	Kedougou	66	64	60	49	48	47
	Sedhiou	68	64	59	42	42	40

WRA, women of reproductive age.

Supplemental Table S19. Risk of high zinc intakes among women of reproductive age and children

		Risk of high intakes (%)					
		Baseline diets	Wheat flour current compliance	Wheat flour improved compliance	Rice	Wheat flour current compliance + rice	Wheat flour improved compliance + rice
		Women of reproductive age					
National	Senegal	0	0	0	0	0	0
Residence	Urban	0	0	0	0	0	0
	Rural	0	0	0	0	0	0
Region	Dakar	0	0	0	0	0	0
	Ziguinchor	0	0	0	0	0	0
	Diourbel	0	0	0	0	0	0
	Saint-Louis	0	0	0	0	0	0
	Tambacounda	0	0	0	0	0	0
	Kaolack	0	0	0	0	0	0
	Thies	0	0	0	0	0	0
	Louga	0	0	0	0	0	0
	Fatick	0	0	0	0	0	0
	Kolda	0	0	0	0	0	0
	Matam	0	0	0	0	0	0
	Kaffrine	0	0	0	0	0	0
	Kedougou	0	0	0	0	0	0
	Sedhiou	0	0	0	0	0	0
		Children 6-59 months					
National	Senegal	0	0	3	33	36	41
Residence	Urban	0	0	4	32	37	43
	Rural	0	1	2	34	36	40
Region	Dakar	0	0	5	34	38	45
	Ziguinchor	1	1	1	46	48	52
	Diourbel	0	0	2	21	24	28
	Saint-Louis	0	0	0	3	5	6
	Tambacounda	2	2	3	42	43	45
	Kaolack	0	0	2	51	52	59
	Thies	0	1	2	20	26	30
	Louga	0	0	1	25	28	32
	Fatick	1	1	3	54	56	62
	Kolda	2	2	5	49	51	57
	Matam	0	0	6	25	28	36
	Kaffrine	0	0	4	45	48	53
	Kedougou	0	0	1	35	37	38
	Sedhiou	0	0	2	56	58	61

WRA, women of reproductive age.

References

Food and Agricultural Organization of the United Nations & World Health Organization (2004). Human energy requirements. In: Report of a Joint FAO/WHO/UNU Expert Consultation. Rome,17–24 October 2001. Rome.

Pyo E., Tsang B.L. & Parker M.E. (2022). Rice as a vehicle for micronutrient fortification: A systematic review of micronutrient retention, organoleptic properties, and consumer acceptability. *Nutr Rev,* 80, 1062-1085. doi: 10.1093/nutrit/nuab107.

USAID Advancing Food Fortification Opportunities to Reinforce Diets (USAID AFFORD) (2023a). Assessing large-scale food fortification opportunities in Senegal. USAID and TechnoServe.

USAID Advancing Food Fortification Opportunities to Reinforce Diets (USAID AFFORD) (2023b). Market assessment of fortified foods in Senegal. USAID and TechnoServe.

World Health Organization (2022). Guideline: Fortification of wheat flour with vitamins and minerals as a public health strategy. Geneva, Switzerland.
